# Triazolopyridopyrimidine: A New Scaffold for Dual-Target Small Molecules for Alzheimer’s Disease Therapy

**DOI:** 10.3390/molecules25143190

**Published:** 2020-07-13

**Authors:** Lazhar Zribi, Irene Pachòn-Angona, Òscar M. Bautista-Aguilera, Daniel Diez-Iriepa, José Marco-Contelles, Lhassane Ismaili, Isabel Iriepa, Fakher Chabchoub

**Affiliations:** 1 Laboratory of Applied Chemistry: Heterocycles, Lipids and Polymers, Faculty of Sciences of Sfax, University of Sfax. B. P 802. 3000 Sfax, Tunisia; zribi.lazhar@gmail.com; 2 Laboratoire de Chimie Organique et Thérapeutique, Neurosciences intégratives et cliniques EA 481, University Bourgogne Franche-Comté, UFR Santé, 19, rue Ambroise Paré, F-25000 Besançon, France; pachon.angona.irene@gmail.com; 3 Department of Organic Chemistry and Inorganic Chemistry, School Sciences, University of Alcalá, Ctra. Barcelona, Km. 33.6, 28871 Alcalá de Henares, Spain; oscar.bautista@uah.es (O.M.B.-A.); daniel.diezi@uah.es (D.D.-I.); 4 Laboratory of Medicinal Chemistry (IQOG, CSIC) C/Juan de la Cierva 3, 28006 Madrid, Spain; jlmarco@iqog.csic.es

**Keywords:** antioxidants, cholinesterase inhibitors, ORAC, triazolopyridopyrimidine

## Abstract

Alzheimer’s disease (AD) is multifactorial disease characterized by the accumulation of abnormal extracellular deposits of amyloid-beta (Aβ) peptide, and intracellular neurofibrillary tangles (NFTs), along with dramatic neuronal death and decreased levels of choline acetyltransferase. Given the limited therapeutic success of available drugs, it is urgent to explore all the opportunities available to combat this illness. Among them, the discovery of new heterocyclic scaffolds binding different receptors involved in AD should offer structural diversity and new therapeutic solutions. In this context, this work describes new triazolopyridopyrimidine easily prepared in good yields showing anticholinesterase inhibition and strong antioxidant power, particularly the most balanced: 6-amino-5-(4-methoxyphenyl)-2-phenyl-[1,2,4]triazolo[1′,5′:1,6] pyrido[2,3-d]pyrimidine-4-carbonitrile(**3c**) with IC_50_ equal to 1.32 μM against AChE and oxygen radical absorbance capacity (ORAC) value equal to 4.01 Trolox equivalents (TE); thus representing a new and very promising hit-triazolopyridopyrimidine for AD therapy.

## 1. Introduction

Alzheimer’s disease (AD) is a neurodegenerative disorder and the most common form of senile dementia, and constitutes one of the major public health problems, mainly due to the increasing old population in developed countries [1,2]. AD is characterized by the progressive loss of cognitive abilities that gradually leads to the physical and mental impairment of patients who eventually die as a consequence of secondary complications associated with the disease, such as pneumopathies, septicemia, infected bedsores or stroke [3]. AD is a multifactorial pathology characterized by two principal hallmarks: the accumulation of abnormal extracellular deposits of amyloid-beta (Aβ) peptide, and intracellular neurofibrillary tangles (NFTs), along with dramatic neuronal death and decreased levels of choline acetyltransferase [4]. Oxidative stress and imbalances in the homeostasis of biometals such as Cu, Fe or Zn [5,6,7] constitute other prominent features of AD pathogenesis. Current AD pharmacotherapy focuses mainly on the impairment of cholinergic and glutamatergic systems, the acetylcholinesterase (AChE) inhibitors donepezil [8], galantamine [9], rivastigmine [8] and memantine, a *N*-methyl-d-aspartate antagonist [8,10], being the only clinically administered drugs, albeit with limited therapeutic success. In view of this situation, new strategies have been promoted based on the multitarget small molecules (MSM) approach [11] for the development of new drugs able to bind simultaneously diverse enzymatic systems or receptors involved in AD pathology [11,12,13,14,15]. Following this paradigm, we developed a number of new MSM [16,17,18] based on azaheterocycle scaffolds. In fact, nitrogen containing heterocycles have been widely used in medicinal chemistry these last years to find new bioactive molecules for AD [19,20,21].

In this context, triazole-containing derivatives have attracted great interest in searching new hit-compounds for AD, as they may display promising in vitro and in vivo AD activities and might be able to prevent drug resistance to certain extent [20,22,23]. Pyridopyrimidines, which are nitrogen bioisosteres of quinoxaline, are associated with a diverse range of biological activities, such as central nervous system (CNS) disorders [24], and constitute a promising scaffold to develop bioactive molecules for AD.

Continuing with our contributions to this area, by using the antioxidant approach associated with cholinesterase (ChE) inhibition based on a new scaffold, we describe here the synthesis of 11 new molecules by combining a triazole ring with a pyridopyrimidine core as well as their cholinesterase inhibition and antioxidant power. Among the 11 synthetized compounds, we identified 6-amino-5-(4-methoxyphenyl)-2-phenyl-[1,2,4]triazolo[1′,5′:1,6]pyrido[2,3-d]pyrimi-dine-4-carbonitrile (**3c**) as the most balanced triazolopyridopyrimidine, exhibiting micromolar inhibition of AChE with IC_50_ equal to 1.32 μM and strong antioxidant activity, representing, thus, a new and very promising hit-triazolopyridopyrimidine for AD therapy.

## 2. Results

### 2.1. Synthesis

**Scheme 1:**
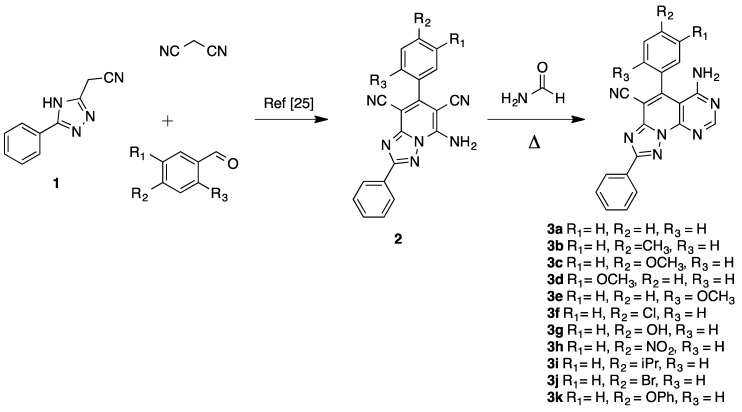
Synthesis of triazolopyridopyrimidine **3a**–**k**.

The synthesis of the new triazolopyridopyrimidine **3a**–**k** has been carried out in a short synthetic procedure as depicted in Scheme 1, in good overall yields, by reacting 2-(5-phenyl-4*H*-1,2,4-triazol-3-yl)acetonitrile (**1**), synthesized from 2-cyanoacetohydrazide and ethyl benzimidate in ethanol at reflux, with the appropriate aldehyde and malonitrile, as reported [25], to afford 5-amino-2-diphenyl-[1,2,4]triazolo[1,5*-a*]pyridine-6,8-dicarbonitriles **2**, followed by treatment with formamide at reflux for 1 h.

**Figure 1:**
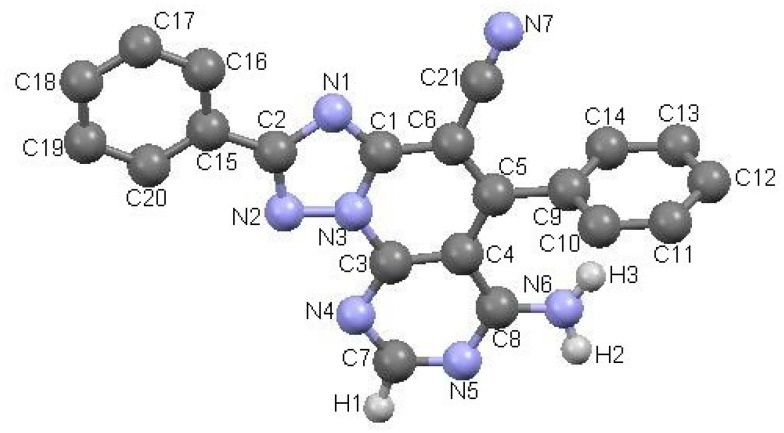
ORTEP (Oak Ridge Thermal-Ellipsoid Plot Program) plot of the X-ray crystal structure of triazolopyridopyrimidine **3a**.

All new triazolopyridopyrimidines showed excellent analytical and spectroscopic data, in good agreement with the expected values (see Section 3 and Supplementary Material). The structure of compound **3a** was also confirmed by X-ray diffraction analysis (Figure 1)

### 2.2. Biological Evaluation

The new compounds were then submitted to antioxidant capacity analysis and cholinesterase inhibition.

#### 2.2.1. Antioxidant Analysis

The ability of triazolopyridopyrimidines **3a**–**k** to reduce the amount of peroxyl radicals was determined by the oxygen radical absorbance capacity by fluorescence (ORAC-FL) method [26,27] using fluorescein (FL) as the fluorescent probe, and 6-hydroxy-2,5,7,8-tetramethyl-chroman-2-carboxylic acid (Trolox) as standard compound. Results were expressed as μmol Trolox equivalents (TE). Melatonin and ferulic acid were used as positive controls, showing ORAC values equal to 2.4 and 3.7 TE, respectively, which fully agree with the values previously described for them [28,29]. As shown in Table 1, all compounds presented a good ability to reduce a peroxyl radical. The ORAC values ranged between 1.77 (**3b)** and 7.17 (**3g**), and seven compounds (**3c**–**e**, **3g**–**h**, **3j**–**k**) showed ORAC values higher than melatonin, whereas three of them (**3c**–**e**) were more potent than ferulic acid. Concerning the structure activity relationship, the most potent antioxidant triazolopyridopyrimidine is **3g**, which bears an hydroxyl group as a substituent at the phenyl ring. Triazolopyridopyrimidines **3c**–**e**, bearing methoxy groups as substituents at the phenyl ring, and **3h** exhibiting a nitro group, are moderate antioxidants. The phenoxy group present in compound **3k** confers weaker antioxidant activity, while methyl (**3b**), chlorine (**3f**), and isopropyl (**3i**) groups do not seem to favor the antioxidant effect, as well as the absence of the substituent as in triazolopyridopyrimidine **3a**.

#### 2.2.2. Inhibition of *Ee*AChE/eqBuChE

For the ChE inhibition experiments we used the cheap and easily available EeAChE and eqBuChE, tacrine as reference, and the Ellman protocol for the determination of the inhibitory potency [30]. All triazolopyridopyrimidines showed a very weak inhibition against BuChE at 10 μM. However, the percentage of inhibition at 10 μM against AChE ranged from 18.17 for **3i** to 73.13 for **3b** (Table 1). The IC_50_ was then evaluated for compounds **3b** and **3c** showing percentages of inhibition at 10 μM higher than 50%. Interestingly these two compounds exhibited an IC_50_ equal to 1.17 μM and 1.32 μM, respectively, only 39- and 44-fold less active than tacrine (IC_50_ = 0.03 μM) [31,32].

### 2.3. Molecular Modeling

To explore the possible binding mode of triazolopyridopyrimidines **3a**–**k** at *Ee*AChE, docking studies were performed for the most promising triazolopyridopyrimidine, **3c**, using AutoDock Vina software [33].

**Figure 2:**
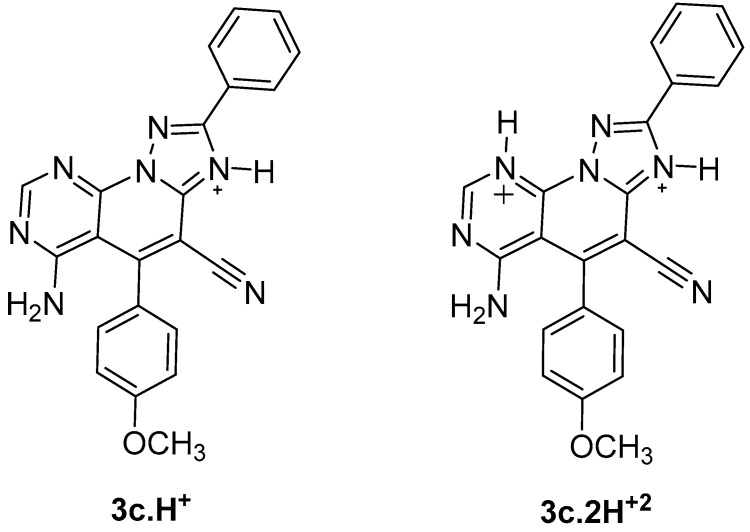
Protonated states of triazolopyridopyrimidine **3c** at physiological pH.

The importance of the role of the protonation state in the ligand-receptor recognition and binding is well known, and consequently, the determination of ligand protonation states was carried out before docking. For compounds **3a**–**k**, the protonated states were computationally predicted at physiological pH. Accordingly, two protonation states were analyzed: protonation of N3 of the triazole ring giving rise to a monoprotonated compound **3c**.H**^+^** and a diprotonated state **3c**.2H^+2^ as a result of the diprotonation of the N3 and N9 of the pyrimidine ring (Figure 2).

Ligand docking studies were performed on the crystal structure of *Ee*AChE taken from Research Collaboratory for Structural Bioinformatics (RCSB) Protein Data Bank (PDB ID: 1C2B, Piscataway, NJ, USA). Docking procedure allows the docking of ligands on the entire protein surface, without prior specification of the binding site (blind docking). The recognition process between ligands and protein was theoretically investigated by flexible docking experiments. At the stage of protein orientation, flexibility in protein structure plays a central role and that is why we usually introduce protein flexibility into the docking procedure. In this case four selected side-chains—Trp286, Tyr124, Tyr337, and Tyr72—were allowed to change their conformations at the same time as the ligand being docked. In this way the access of bulky ligands, such as triazolopyridopyrimidine **3c**, to the catalytic site can be facilitated. Inhibitors were analyzed on the basis of their affinity energy and type of interactions that they made with protein residues.

**Figure 3:**
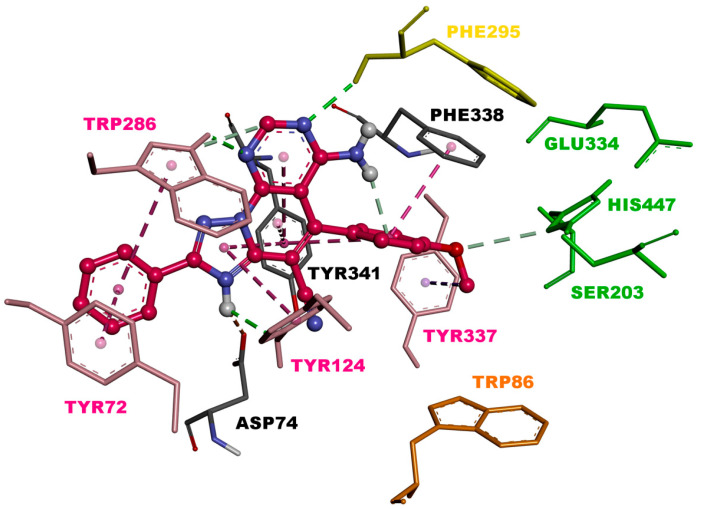
Binding mode of inhibitor **3c**.H^+^ within the active site of *Ee*AChE (binding affinity: –11.5 kcal/mol). Compound **3c**.H^+^ is illustrated in red. Ligand is rendered as ball and sticks and the side chain conformations of the mobile residues are illustrated in the same color as the ligand. Catalytic triad amino acids (CT) are in green, anionic sub-site (AS) in orange, acyl binding pocket (ABP) in yellow, and peripheral anionic subsite (PAS) in light red, and color coded.

**Figure 4:**
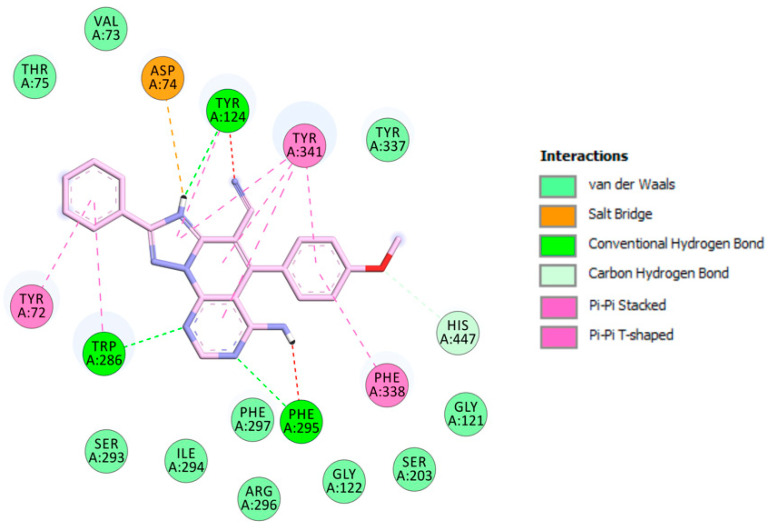
Schematic 2D representation of interactions of **3c**.H^+^ with *Ee*AChE.

The visualization of 3c.H^+^ inside *Ee*AChE enzyme revealed several important interactions. This triazolopyridopyrimidine simultaneously occupied both the catalytic anionic site (CAS) and peripheral anionic subsite (PAS) of AChE (Figure 3). The methoxybenzene ring was located at the CAS through carbon hydrogen interaction with the amino acid of the catalytic triad, His447. Additionally, the methoxybenzene ring formed a π-π stacked interaction with Phe338 and a T-shaped interaction with Tyr341 (Figure 4). In the middle of the gorge, the amino acids, Tyr341 and Tyr124, were also observed to form π-π stacked and T-shaped interactions with fused-rings part of the envisioned triazolopyridopyrimidine. The triazolopyridopyrimidine moiety forms a stable network of hydrogen bonding between the protonated N3 and N9 with Tyr124 and Phe295, respectively (Figure 4). The phenyl ring is located in the pocket forming PAS interacting with Tyr72 and Trp286 via face-to-face and edge-to-face π-π interactions, respectively. In this situation, Trp286 also established a hydrogen bond with a pyrimidine nitrogen. The protonated triazole moiety interacted with Asp74, in PAS, via salt bridge interaction (Figure 4).

For compounds **3a**.H^+^–**3k**.H^+^ AutoDock Vina runs resulted in binding energy scores that ranged from −8.7 to −12.1 kcal/mol. Although there was no exact correlation between docking score and inhibitory activity, we found some correlation between experimental activities and computational binding energies. Among the 11 triazolopyridopyrimidines, the less active compounds, **3i**.H^+^ and **3k**.H^+^, showed the highest binding energy values at −8.7 and −9.6 kcal/mol, respectively. The most active compound was found to be **3b**.H^+^ and it exhibited the lowest dock score of −12.1 kcal/mol. The second most active compound, **3c**.H^+^, exhibited a dock score of −11.5 kcal/mol. The rest of the compounds (**3a**.H^+^, **3d**.H^+^–**3h**.H^+^, and **3j**.H^+^) have shown binding energy scores that ranged from −9.6 to −11.5 kcal/mol, but correlation with percentage of inhibition seems hard to establish.

**Figure 5:**
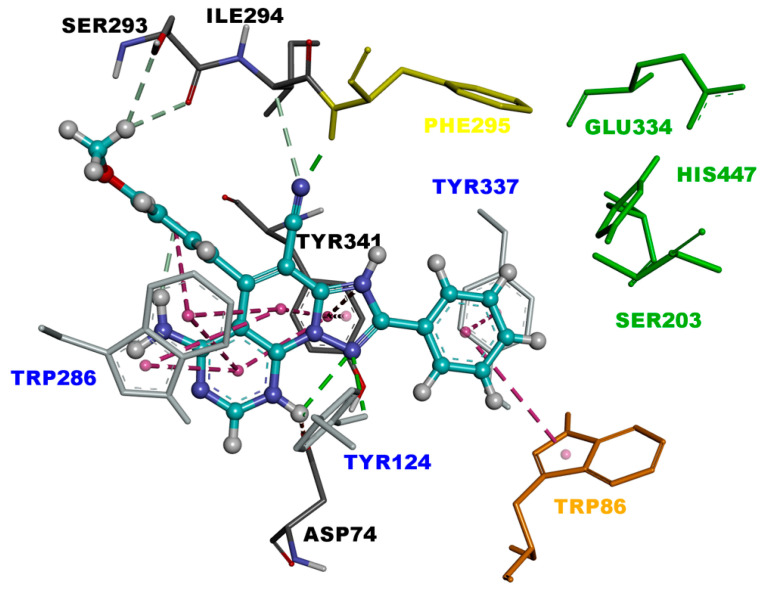
Binding mode of inhibitor **3c**.2H^+2^ (blue) at the active site of *Ee*AChE. Ligand is rendered as ball and sticks, and the side chain conformations of the mobile residues are illustrated in the same color as the ligand. Catalytic triad (CT) is colored in green, anionic sub-site (AS) in orange, acyl binding pocket (ABP) in yellow, and peripheral anionic subsite (PAS) in light blue, and color coded.

**Figure 6:**
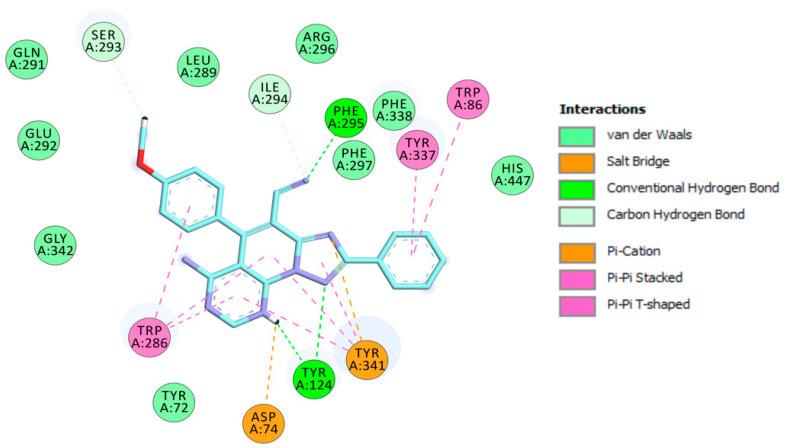
Schematic 2D representation of different interactions of **3c**.2H^+2^ at *Ee*AChE.

Triazolopyridopyrimidine **3c**.2H^+2^ is bound to the *Ee*AChE active site in similar fashion as **3c**.H^+^, with a binding affinity of −10.4 kcal/mol. As shown in Figure 5, triazolopyridopyrimidine **3c**.2H^+2^ is located in the entire enzymatic gorge. Docking simulations showed a clear preference to accommodate the pyrimidine and methoxyphenyl moieties within the PAS. The phenyl ring is embedded in the binding pocket forming the CAS, with the pyridotriazole moiety lying in the middle of the gorge between the CAS and PAS. In the complex, the phenyl ring points towards the catalytic triad residues (His447, Ser203, and Glu334) and interacts via van der Waals interactions with His447. It was also found that this ring forms π-π T-shaped and π-stacked interactions with Trp86 and Tyr337 (Figure 5 and Figure 6).

The methoxyphenyl moiety facilitates π-π T-shaped interaction with the side chain of Trp286 from the PAS at the gorge opening (Figure 6). Similarly, the pyrimidine and pyridine motifs provide π-π stacked interactions with Trp286. Furthermore, the triazolopyridopyrimidine moiety provides π-π stacked interactions with Tyr341. In addition, protonated nitrogen facilitates salt bridge and π-cation interactions with Asp74 and Tyr341. Finally, three hydrogen bond interactions with Tyr124 and Phe295 strengthen the protein–ligand interaction (Figure 6).

**Figure 7:**
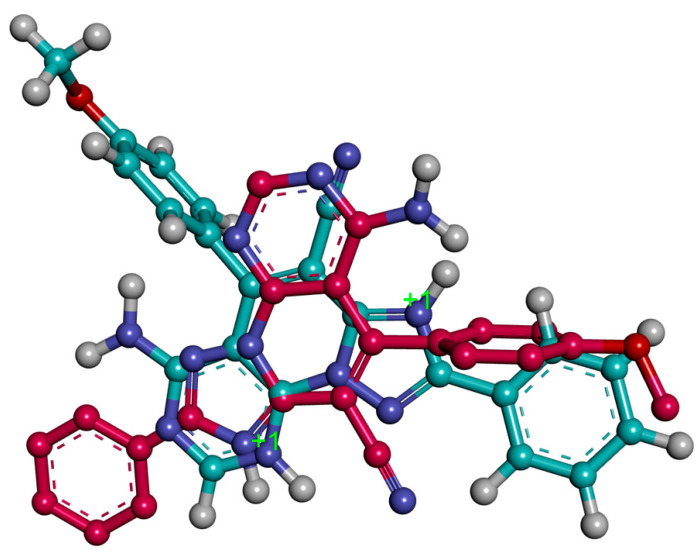
Superposition of bioactive conformations for **3c**.H^+^ (red) and **3c**.2H^+2^ (blue).

The most stable conformation generated through the docking studies for both **3c**.H^+^ and **3c**.2H^+2^ showed that these ligands occupy the same spatial region in the AChE enzyme, but the arrangement of the three-ring system is different. They share the position of the central core of the ligands (pyridine moieties) and this implies that the phenyl moiety of **3c**.2H^+2^ occupies a similar position as the methoxyphenyl moiety of the **3c**.H^+^ (Figure 7). The binding modes place the ligands interacting with both the PAS and the CAS.

## 3. Materials and Methods

All reagents were purchased from Sigma Aldrich. Melting points were determined on a Kofler apparatus (Wagner Munz, München, Germany). Progress of the reactions was monitored with Thin-layer chromatography (TLC) using aluminum sheets with silica gel 60 F254 from Merck (Kenilworth, NJ, USA). ^1^H and ^13^C-NMR spectra were recorded on a Bruker spectrometer, operating at 400 and 100 MHz, respectively, using DMSO-*d*_6_ as solvent. The chemical shifts were reported in parts per million (ppm), using tetramethylsilane (**TMS**) as internal reference. The multiplicities of the signals are indicated by the following abbreviations: s, singlet; d, doublet; t, triplet; q, quadruplet; and m, multiplet; coupling constants are expressed in Hz. Elemental analyses were obtained by a Carlo Erba EA 1108 analyzer and the analytical results were within ± 0.2% of the theoretical values for all compounds.

### 3.1. Synthesis of Compounds ***3a–k***

In a typical experiment, a solution of 5-amino-2-diphenyl-[1,2,4]triazolo[1,5*-a*]pyridine-6,8-dicarbonitrile (0.01 mol) and formamide (7 mL, Aldrich, St. Quentin Fallavier, France) was heated under reflux for 1 h. The solid product formed by cooling was filtered, washed with water and ethyl acetate, and dried. Following this protocol, it was possible to synthesize 6-amino-5-(aryl)-2-phenyl-[1,2,4]triazolo[1′,5′:1,6] pyrido[2,3-d]pyrimidine-4-carbonitrile **1**–**11** with good yields.

*6-Amino-2,5-diphenyl-*[1,2,4]*triazolo[1′,5′:1,6]pyrido[2,3-d] pyrimidine-4-carbonitrile***3a**, Rdt = (76%); mp > 260 °C; ^1^H-NMR (400 MHz, DMSO*d*_6_) δ 8.70 (s, 1H), 8.57 (s, 1H, NH), 8.29–8.29 (m, 2H), 7.76–7.73 (m, 3H), 7.65 (m, 2H), 7.61–7.59 (m,3H), 4.90 (s, 1H, NH), ^1^^3^C-NMR (100 MHz, DMSO*d*_6_) δ 164.5, 162.3, 160.4,150.6, 149.8, 149.6, 135.2, 131.3, 131.2, 130.4, 130.0, 129.6, 128.6, 127.6, 114.4, 99.9, 98.9. Anal. Calcd: for C_21_H_15_N_7_: C, 69.03; H, 4.14; N, 26.83 found: C, 69.75; H, 3.58; N, 26.67.

*6-Amino-2-phenyl-5-(p-tolyl)-*[1,2,4]*triazolo[1′,5′:1,6]pyrido[2,3-d] pyrimidine-4-carbonitrile***3b,** Rdt = (74%); mp > 260 °C; ^1^H-NMR (400 MHz, DMSO*d*_6_) δ 8.67 (s,1H), 8.54 (s, 1H, NH), 8.27 (d, *J* = 3,2 Hz, 2H), 7.58–7.47 (m, 7H), 4.98 (s, 1H, NH), 2.48 (s, 3H, CH_3_), ^1^^3^C-NMR (100 MHz, DMSO*d*_6_) δ 164.4, 162.4, 160.4, 150.5, 150.0, 149.4, 141.0, 132.3, 131.3, 131.0, 130.0, 129.6, 128.5, 127.6, 114.4, 100.0, 98.0, 21.5 Anal. Calcd: for C_22_H_17_N_7_ C, 69.64; H, 4.52; N, 25.84 found: C, 69.85; H, 4.08; N, 26.07.

*6-Amino-5-(4-methoxyphenyl)-2-phenyl-*[1,2,4]*triazolo[1′,5′:1,6]pyrido[2,3-d] pyrimidine-4-carbonitrile***3c,** Rdt = (75%); mp > 260 °C; ^1^H-NMR (400 MHz, DMSO*d*_6_) δ 8.66 (s,1H), 8.54 (s, 1H, NH), 8.27 (2H, d, *J* = 6,9 Hz), 7.59–7.57 (m, 5H), 7.29–7.26 (d, 2H, *J* = 6,9 Hz), 5.09 (s, 1H, NH), 3.91 (3H, s, OCH_3_), ^1^^3^C-NMR (100 MHz, DMSO*d*_6_) δ 164.4, 162.4, 161.3, 160.3, 150.6, 149.9, 149.4, 131.3, 130.3, 130.0, 129.6, 127.6, 126.8, 115.8, 114.5, 100.1, 99.0, 55.9. Anal. Calcd: for C_22_H_17_N_7_O C, 66.82; H, 4.33; N, 24.80; found: C, 67.44; H, 3.85; N, 24.70.

*6-Amino-5-(3-methoxyphenyl)-2-phenyl-*[1,2,4]*triazolo[1′,5′:1,6]pyrido[2,3-d] pyrimidine-4-carbonitrile***3d,** Rdt = (73%); mp > 260 °C; ^1^H-NMR (400 MHz, DMSO*d*_6_) δ 8.67 (s, 1H), 8.57 (s, 1H, NH), 8.36–8.26 (m, 2H), 7.71–7.31 (m, 7H), 5.19 (s, 1H), 3.79 (s, 3H), ^1^^3^C-NMR (100 MHz, DMSO*d*_6_) δ 164.5, 162.4, 160.4, 156.1,150.6,149.5,147.0, 133.5, 131.3, 130.0, 129.9, 129.6, 127.6,123.2, 122.4, 114.3, 113.4, 100.1, 99.1, 56.6 Anal. Calcd: for C_22_H_17_N_7_O: C, 66.82; H, 4.33; N, 24.80; found: C, 67.26; H, 3.79; N, 25.15.

*6-Amino-5-(2-methoxyphenyl)-2-phenyl-*[1,2,4]*triazolo[1′,5′:1,6]pyrido[2,3-d] pyrimidine-4-carbonitrile***3e,** Rdt = (70%); mp > 260 °C; ^1^H-NMR (400 MHz, DMSO*d*_6_) δ 8.69 (s,1H), 8.55 (s,1H, NH), 8.27–8.29 (m, 2H), 7.71–7.75(m, 1H), 7.56 (m, 4H), 7.39–7.42 (m, 1H,), 7.29–7.32 (m, 1H), 5.2 (s,1H, NH), 3.81 (s, 3H, OCH_3_), ^1^^3^C-NMR (100 MHz, DMSO*d*_6_) δ 164.5, 162.5, 160.4, 156.1, 150.6,149.5,147.0, 133.5, 131.3, 130.1, 130.0, 129.6, 127.6,123.3, 122.4, 114.3, 113.4, 100.1, 99.2, 56.6 Anal. Calcd: for C_22_H_17_N_7_O: C, 66.82; H, 4.33; N, 24.80; O, 4.05 found: C, 67.47; H, 3.88; N, 25.08.

*6-Amino-5-(4-chlorophenyl)-2-phenyl-*[1,2,4]*triazolo[1′,5′:1,6]pyrido[2,3-d] pyrimidine-4-carbonitrile***3f,** Rdt = (74%); mp > 260 °C; ^1^H-NMR (400 MHz, DMSO*d*_6_) δ 8.70 (s,1H), 8.54 (s, 1H, NH), 8.29 (m, 2H), 7.81 (m, 1H,), 7.69–7.56 (m, 6H), 5.19 (s,1H, NH), ^1^^3^C-NMR (100 MHz, DMSO*d*_6_) δ 164.5, 162.3, 160.3,150.6,149.6,148.8,136.1, 133.8, 131.4, 130.9,130.5, 130.0, 129.6, 127.6, 114.2, 99.8, 99.0. Anal. Calcd: for C_21_H_14_ClN_7_: C, 63.08; H, 3.53; N, 24.52 found: C, 63.57; H, 3.01; N, 24.58.

*6-Amino-5-(4-hydroxyphenyl)-2-phenyl-*[1,2,4]*triazolo[1′,5′:1,6]pyrido[2,3-d] pyrimidine-4-carbonitrile***3g,** Rdt = (69%); mp > 260 °C; ^1^H-NMR (400 MHz, DMSO*d*_6_) δ 10.27 (s, 1H,OH) 8.66 (s, 1H), 8.54 (s, 1H, NH), 8.30–8.25 (m, 2H), 7.57–7.54 (m, 3H), 7.43 (d, *J* = 8,3 Hz, 2H), 7.08 (d, *J* = 8,3 Hz, 2H), 5.15 (s, 1H, NH) ^1^^3^C-NMR (100 MHz, DMSO*d*_6_) δ 164.4, 162.5, 160.3, 159.9, 150.6,150.4,149.4, 131.3, 130.2,130.0,129.6, 127.6,125.1, 117.1, 114.6, 100.2, 99.0 Anal. Calcd: for C_21_H_15_N_7_O: C, 66.13; H, 3.96; N, 25.71; O, 4.19 found: C, 66.59; H, 3.43; N, 25.95.

*6-Amino-5-(4-nitrophenyl)-2-phenyl-*[1,2,4]*triazolo[1′,5′:1,6]pyrido[2,3-d] pyrimidine-4-carbonitrile***3h,** Rdt = (71%); mp > 260 °C; ^1^H-NMR (400 MHz, DMSO*d*_6_) δ 8.68 (s, 1H), 8.42 (s, 1H, NH), 8.27 (s, 1H), 7.93 (s, 1H), 7.61 (d, J = 8,1 Hz, 1H), 7.58 (m, 5H), 5.12(s, 1H,NH), ^1^^3^C-NMR (100 MHz, DMSO*d*_6_) δ 164.5, 162.4, 160.7, 160.3, 150.6,149.6,149.5, 140.5, 131.3, 130.0, 129.6, 129.5, 129.4, 127.6, 120.5, 114.4, 99.9, 99.0. Anal. Calcd: for C_21_H_12_N_8_O_2_: C, 61.76; H, 2.96; N, 27.44 found C, 61.67; H, 3.01; N, 27.38.

*6-Amino-5-(4-isopropylphenyl)-2-phenyl-*[1,2,4]*triazolo[1′,5′:1,6]pyrido[2,3-d] pyrimidine-4-carbonitrile***3i,** Rdt = (76%); mp > 260 °C; ^1^H-NMR (400 MHz, DMSO*d*_6_) δ 8.68 (s, 1H), 8.51 (s, 1H, NH), 8.30 (m, 2H), 7.63–7.55 (m, 7H), 5.93(s, 1H,NH), 3.07(m, 1H, CH), 1.32(s, 3H, CH3), 1.30(s, 3H,CH3), ^1^^3^C-NMR (100 MHz, DMSO*d*_6_) δ 164.5, 162.4, 160.4, 151.7, 150.6, 150.0, 149.5, 132.6, 131.3,130.0,129.6, 128.6,128.3, 127.6, 124.9, 114.5, 100.0, 98.9, 33.8, 24.1. Anal. Calcd: for C_24_H_19_N_7_:C, 71.09; H, 4.72; N, 24.18 found C, 70.67; H, 4.61; N, 24.26.

*6-Amino-5-(4-bromophenyl)-2-phenyl-*[1,2,4]*triazolo[1′,5′:1,6]pyrido[2,3-d] pyrimidine-4-carbonitrile***3j:** Rdt = (74%); mp > 260 °C; ^1^H-NMR (400 MHz, DMSO*d*_6_) δ 8.67 (s, 1H), 8.49 (s, 1H, NH), 8.26–8.28 (m, 2H), 7.93–7.95 (d, J = 8,4 Hz, 2H), 7.60 7.63(d, J = 8,4 Hz, 2H), 7.58 (m, 3H), 5.16 (s, 1H, NH), ^1^^3^C-NMR (100 MHz, DMSO*d*_6_) δ 164.5, 162.2, 160.3, 150.5, 150.6, 1549.5, 148.7, 134.2, 133,4, 131.4, 131.0, 129.9, 129.6, 127.6, 124.9, 114.3, 99.7, 98.9. Anal. Calcd: for C_21_H_12_BrN_7_: C, 57.03; H, 2.73; Br, 18.07; N, 22.17 found C, 57.89; H, 2.68; N, 22.23.

*6-Amino-5-(4-phenoxyphenyl)-2-phenyl-*[1,2,4]*triazolo[1′,5′:1,6]pyrido[2,3-d] pyrimidine-4-carbonitrile***3k,** Rdt = (67%); mp > 260 °C; ^1^H-NMR (400 MHz, DMSO*d*_6_) δ 8.65 (s, 1H), 8.53 (s, 1H, NH), 8.26 (s, 2H), 7.66 (m, 2H), 7.56 (s, 2H), 7.52–7.49 (m, 2H), 7.33–7.24 (m, 3H), 7.19 (m, 3H), 5.20(s, 1H,NH), ^1^^3^C-NMR (100 MHz, DMSO*d*_6_) δ 164.4, 162.3, 160.3, 159.8, 155.8, 150.5, 149.5, 130.9, 130.8, 130.7, 130.0, 129.5, 129.3, 127.6, 125.0, 120.1, 119.9, 119.4, 114.4, 99.9, 99.0. Anal. Calcd: for C_27_H_17_N_7_O: C, 71.20; H, 3.76; N, 21.53; O, 3.51 found C, 70.99; H, 3.81; N, 21.66.

### 3.2. Biological Evaluation

#### 3.2.1. Oxygen Radical Absorbance Capacity Assay

The antioxidant activity of compounds **3a**–**k** was carried out by ORAC-FL using fluorescein as a fluorescent probe. Briefly, fluorescein and antioxidant were incubated in a black 96-well microplate (Nunc) for 15 min at 37 °C. 2,2′-Azobis(amidinopropane) dihydrochloride was then added quickly using the built-in injector of Varioskan Flash plate reader (Thermo Scientific). The fluorescence was measured at 485 nm (excitation wavelength) and 535 nm (emission wavelength) each min for 1 h. All the reactions were made in triplicate and at least three different assays were performed for each sample.

#### 3.2.2. Inhibition of *Ee*AChE and eqBuChE

The effect of the test compounds on the activity of both *Ee*AChE and eqBuChE was evaluated following the spectrophotometric method of Ellman [30]. The reaction occurred in a final volume of 3 mL of a 0.1 M phosphate-buffered solution at pH = 8.0, containing 5,5′-dithiobis-2-nitrobenzoic acid (DTNB, 2625 µL, 0.35 mM, final concentration), *Ee*AChE (29 µL, 0.035 U/mL, final concentration) or eqBuChE (60 µL, 0.05 U/mL, final concentration), tested compound (3 µL, 0.001–1000 nM, final concentrations), and 1% *w*/*v* bovine albumin serum phosphate-buffered (pH = 8) solution (BSA, 60 µL). After this pre-incubation period, acetylthiocholine iodide (105 µL, 0.35 mM, final concentration) or butyrylthiocholine iodide (150 µL, 0.5 mM, final concentration) was added, allowing 15 min of additional incubation time. For IC_50_, inhibition curves were built by pre-incubating this blend at room temperature with nine concentrations of each compound for 10 min.

#### 3.2.3. Molecular Modeling

To carry out docking simulations, the starting ligand geometries for compounds **3c**.H^+^ and (**3c.2H**)^2+^ were built with Discovery Studio (DS, San Diego, CA, USA), version 2.1, software package and then protonated at pH 7.4. The resulting molecules were subsequently minimized using the adopted-based Newton–Rapson algorithm. Structure was considered fully optimized when the energy changes between interactions were less than 0.01 kcal/mol [34]. The coordinates of *Ee*AChE (PDB:1C2B) were obtained from the Protein Data Bank (PDB). The structure was initially processed by “prepare protein” module in DS, to give the structure suitable for docking. Missed side chains of the protein were added and the water molecules were removed; later the structure was protonated at pH 7.4. AutoDockTools (ADT; version 1.5.4, La Jolla, CA, USA) was used to add hydrogens and partial charges for proteins and ligands using Gasteiger charges. Flexible torsions in the ligands were assigned with the AutoTors module, and the acyclic dihedral angles were allowed to rotate freely. Docking runs were carried out allowing the rotation of Trp286, Tyr124, Tyr337, and Tyr72 receptor residues, using the AutoTors module.

Ligand–protein docking was conducted using the program Autodock Vina [33]. The box center was defined, and the docking box was displayed using ADT. The docking procedure was applied to the whole protein target, without imposing the binding site (“blind docking”). The coordinates of the grid box were as follows: x = 21.5911, y = 87.752, and z = −23.591. The grid dimensions were as follows: x = 60, y = 60, and z = 72, using a spacing of 1 Ǻ, and a num_modes of 40. The top ranked conformations for each docked compound were retained and visually inspected for binding pattern analysis, which was visualized and depicted in DS software.

## 4. Conclusions

AD is a neurodegenerative disorder and the most common form of senile dementia. The four current AD pharmacotherapies focus on the impairment of cholinergic and glutamatergic, but with limited therapeutic success. Therefore, it is urgent to explore all the opportunities in order to combat this illness, such as the discovery of new heterocyclic scaffolds binding with different receptors involved in AD to offer more structural diversity and new solutions in this field. For this purpose, in this work we developed new triazolopyridopyrimidines easily prepared in three steps in good yields showing antioxidant activity and inhibition of AChE. As a result, we identified 6-amino-5-(4-methoxyphenyl)-2-phenyl-[1,2,4]triazolo[1′,5′:1,6]pyrido[2,3-d] pyrimidine-4-carbonitrile (**3c**) as the most balanced compound showing micromolar inhibition of AChE with IC_50_ equal to 1.3 μM. Molecular modeling of monoprotonated and diprotonated states of **3c** revealed several important interactions with AChE and showed that **3c.H**^+^ simultaneously occupied both the CAS and PAS of AChE while **3c.2H**^2+^ bound principally at active site and was located in the entire enzymatic gorge. This new type of heterocycle, and particularly triazolopyridopyrimidine **3c**, constitute very promising findings that deserve further investigation for AD. Work is now in progress in our laboratories to develop analogues with the best pharmacological profile by exploring the impact to introduce different substituents on the aromatic ring attached to the triazolo core. The investigation of the activities of these new compounds as inhibitors of Aβ self-aggregation will be explored too. The results will be reported in due course.

## Figures and Tables

**Table 1:**
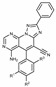
Inhibition percentages for triazolopyridopyrimidines **3a**–**k** at 10 μM against EeAChE and their oxygen radical absorbance capacity (ORAC) (Trolox equivalents, TE) values.

Compound	R^1^	R^2^	R^3^	ORAC ^a^ (TE)	% Inhibition of EeAChE ^b^ at 10 μM	IC_50_ (μM) ^c^
3a	H	H	H	1.85 ± 0.1	40.27 ± 2.4	nd
3b	H	CH_3_	H	1.77 ± 0.1	73.13 ± 0.1	1.17 ± 0.0
3c	H	OCH_3_	H	4.01 ± 0.1	63.93 ± 1.8	1.32 ± 0.0
3d	OCH_3_	H	H	4.33 ± 0.1	31.23 ± 2.1	nd
3e	H	H	OCH_3_	3.79 ± 0.1	32.33 ± 2.4	nd
3f	H	Cl	H	1.81 ± 0.1	43.90 ± 1.2	nd
3g	H	OH	H	7.17 ± 0.2	36.43 ± 0.6	nd
3h	H	NO_2_	H	3.54 ± 0.1	44.63 ± 1.6	nd
3i	H	iPr	H	1.89 ± 0.1	18.17 ± 1	nd
3j	H	Br	H	2.57 ± 0.0	38.13 ± 1.1	nd
3k	H	OPh	H	2.99 ± 0.2	18.60 ± 1.8	nd
Melatonin	-	-	-	2.45 ± 0.1	-	-
Ferulic acid	-	-	-	3.74 ± 0.2	-	-
Tacrine	-	-	-		95.63 ± 2.1	0.03 ± 0.0

^a^ Data are expressed as Trolox equivalents and are the mean (*n* = 3) ± SEM. ^b^ Every percentage value is the mean of a triple of at least two different experiments. ^c^ Each IC_50_ value is the mean ± SEM of a quadruplicate of at least three different experiments; nd: not determined.

## Supplementary Materials

The following are available online: NMR spectra of compounds **3a–k**.
