# Supplementary material for: Triazolopyridopyrimidine: A New Scaffold for Dual-Target Small Molecules for Alzheimer’s Disease Therapy
Source: Molecules. 2020 Jul 13;25(14):3190. doi: 10.3390/molecules25143190 (PMC7397274; doi:10.3390/molecules25143190)
Supplement: Supplementary file 1 [file molecules-25-03190-s001.pdf]

Triazolopyridopyrimidine: A New Scaffold for Dual-Target Small Molecules for Alzheimer's Disease Therapy

Lazhar Zribi [1], Irene Pachòn-Angona [2], Òscar M. Bautista-Aguilera [3], Daniel Diez-Iriepa [3], José Marco-Contelles [4], Lhassane Ismaili [2,*], Isabel Iriepa [3,*] and Fakher Chabchoub [1,*]

[1] Laboratory of Applied Chemistry: Heterocycles, Lipids and Polymers, Faculty of Sciences of Sfax, University of Sfax. B. P 802. 3000 Sfax-Tunisia; zribi.lazhar@gmail.com

[2] Laboratoire de Chimie Organique et Thérapeutique, Neurosciences intégratives et cliniques EA 481, Univ. Bourgogne Franche-Comté, UFR Santé, 19, rue Ambroise Paré, F-25000 Besançon, France; pachon.angona.irene@gmail.com

[3] Department of Organic Chemistry and Inorganic Chemistry, School Sciences, University of Alcalá, Ctra. Barcelona, Km. 33.6, 28871, Alcalá de Henares, Spain; oscar.bautista@uah.es (O.M.B.-A.); daniel.diezi@uah.es (D.D.-I.)

[4] Laboratory of Medicinal Chemistry (IQOG, CSIC) C/Juan de la Cierva 3, 28006-Madrid, Spain; jlmarco@iqog.csic.es

* Correspondence: lhassane.ismaili@univ-fcomte.fr (L.I.); isabel.iriepa@uah.es (I.I.); fakher.chabchoub@yahoo.fr (F.C.)

CONTENT

NMR spectra of compounds **3a-k**…………………………………………………........S2-S13

^{1}H NMR of compound **3a** (400 MHz, DMSO*d6*)

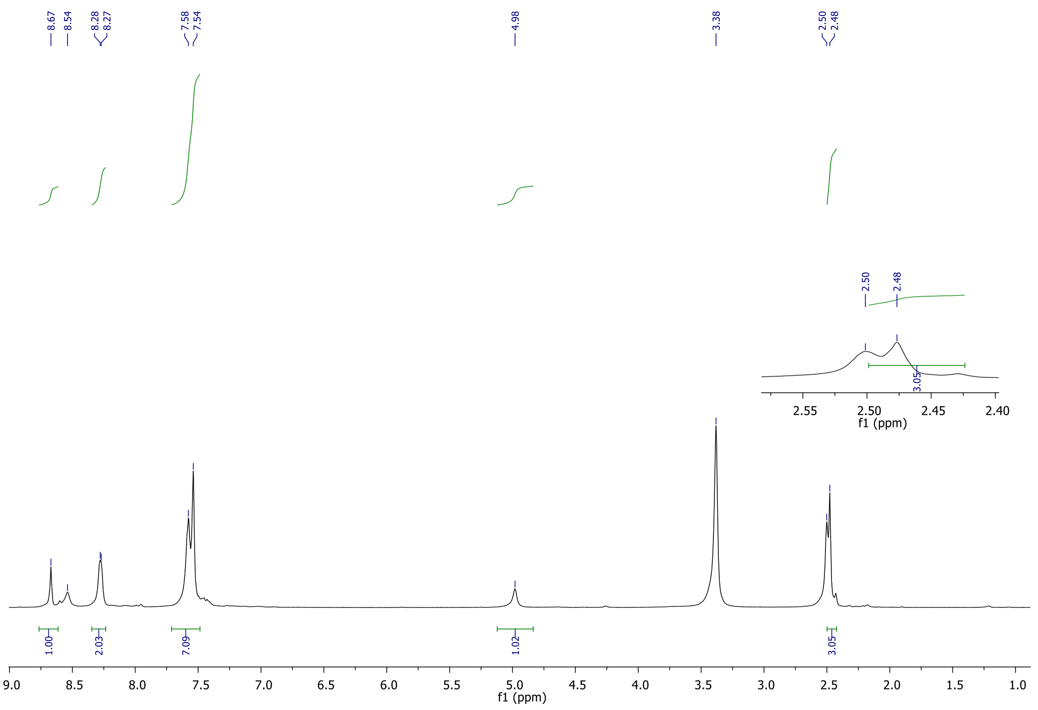

^{13}C NMR of compound **3a** (100 MHz, DMSO*d6*)

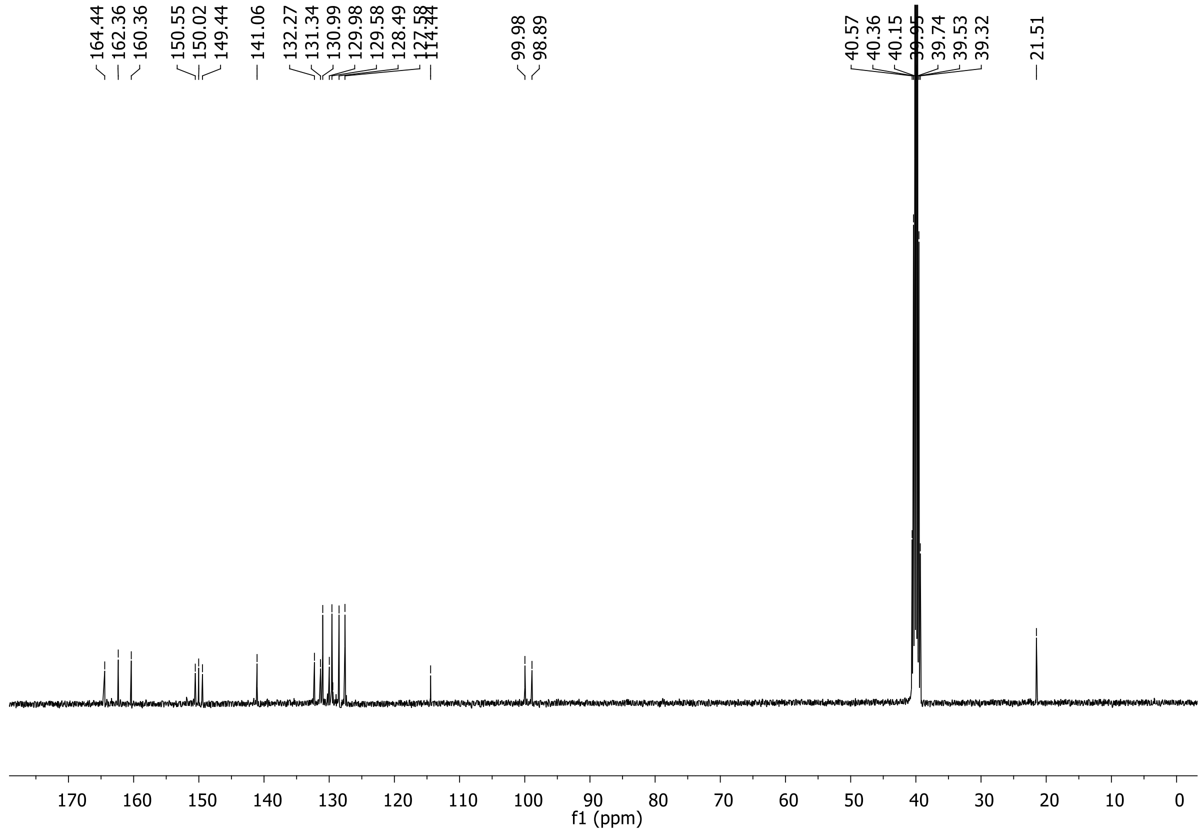

2D **HSQC** spectrum of compound **3a**

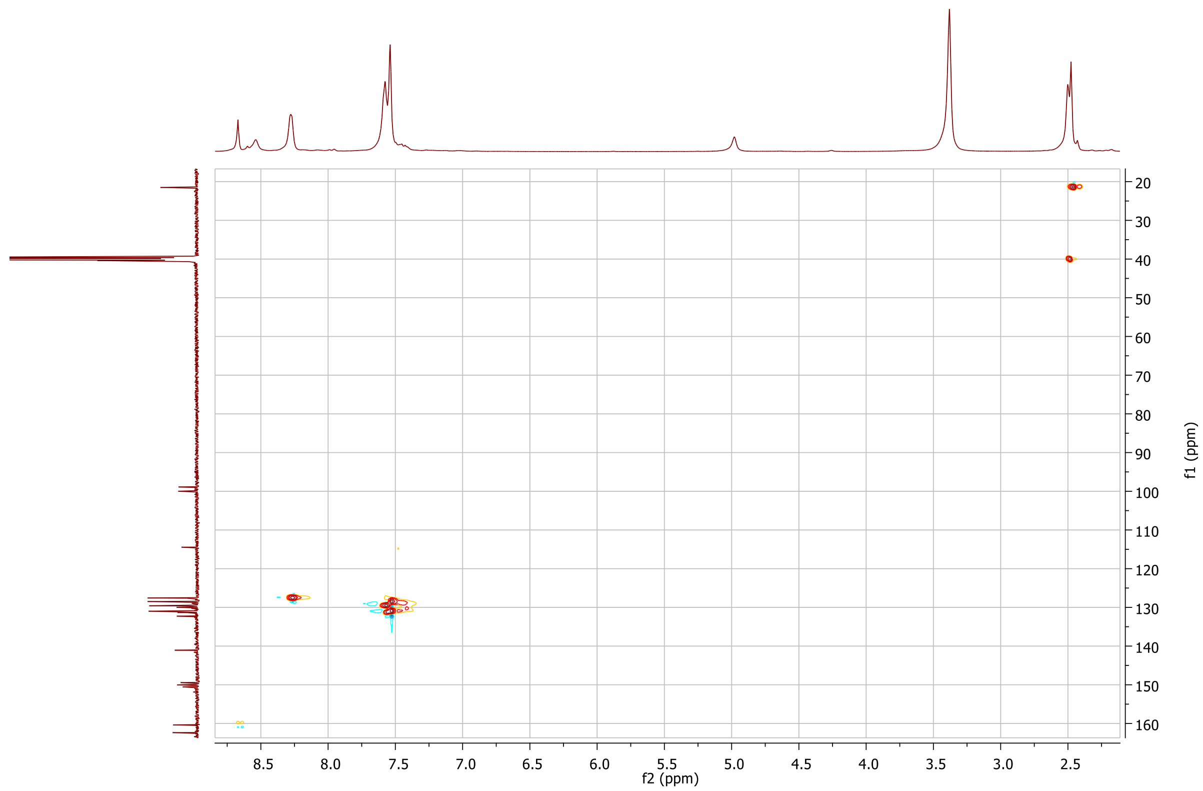

2D **HMBC** spectrum of compound **3a**

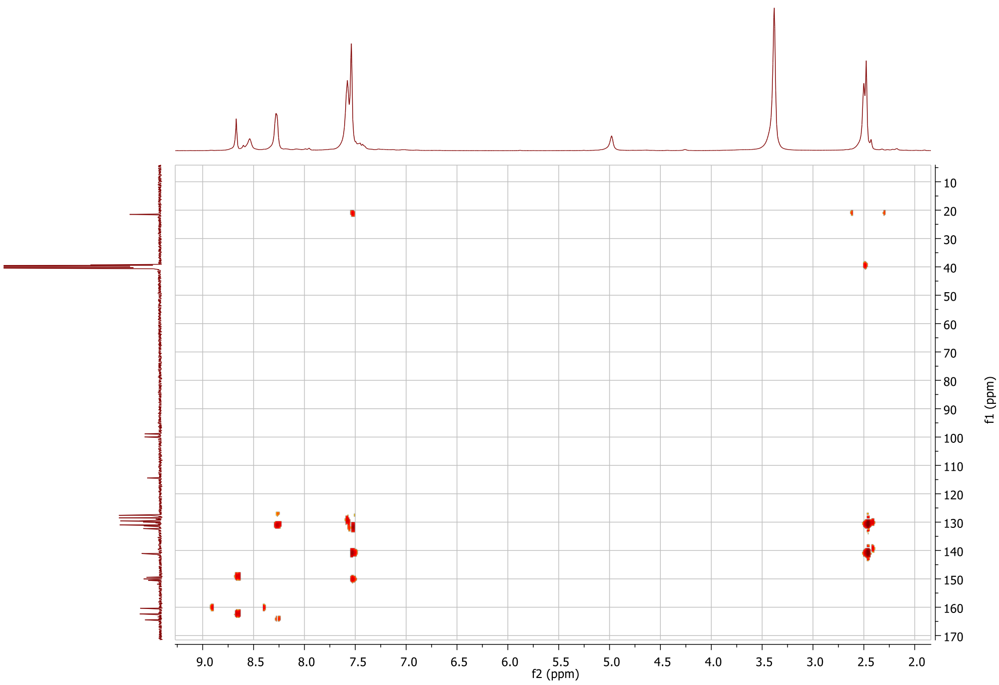

^{1}H NMR of compound **3b** (400 MHz, DMSO*d6*)

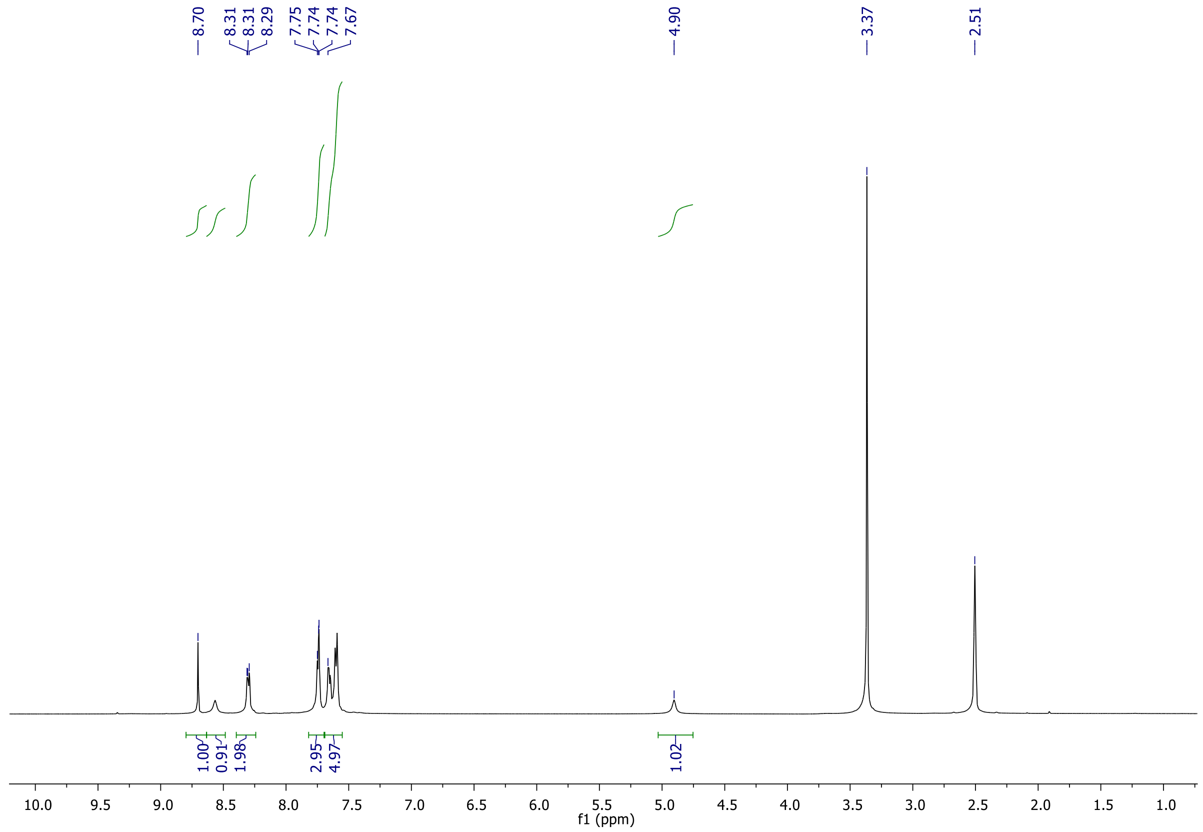

^{13}C NMR of compound **3b** (100 MHz, DMSO*d6*)

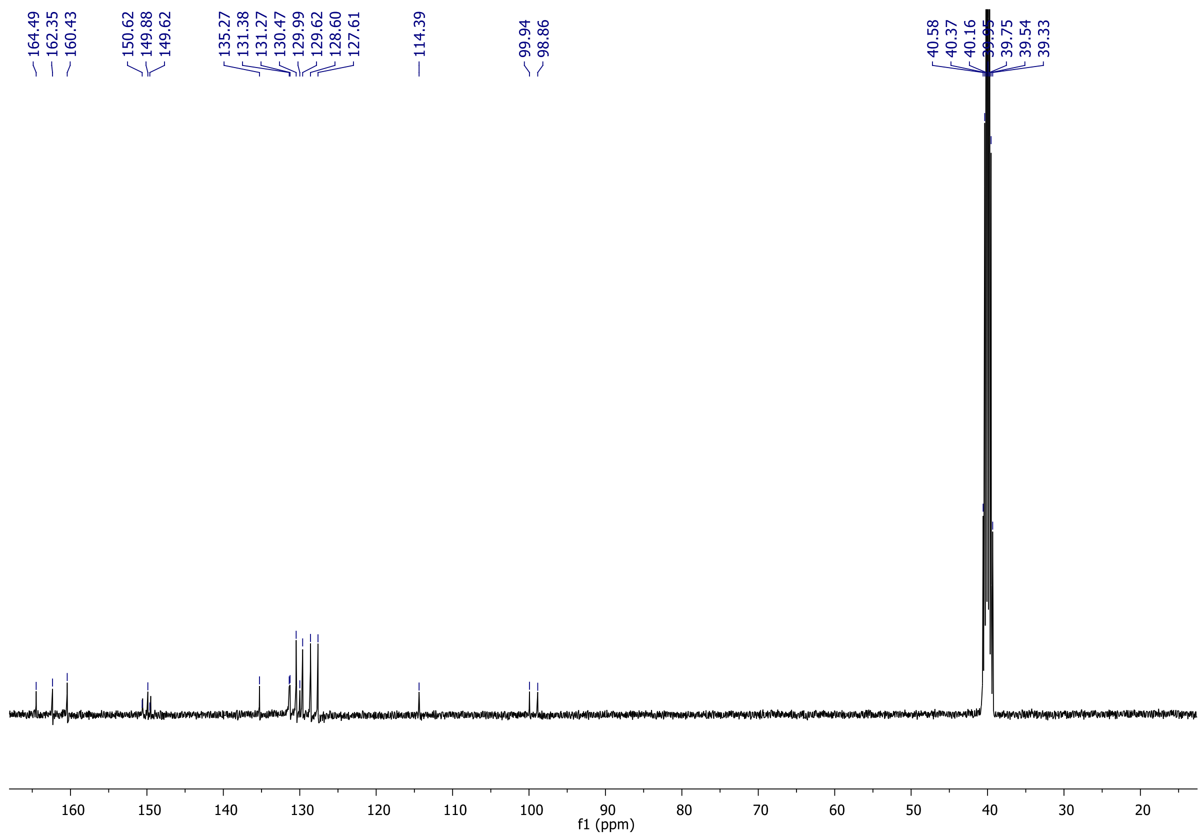

^{1}H NMR of compound **3c** (400 MHz, DMSO*d6*)

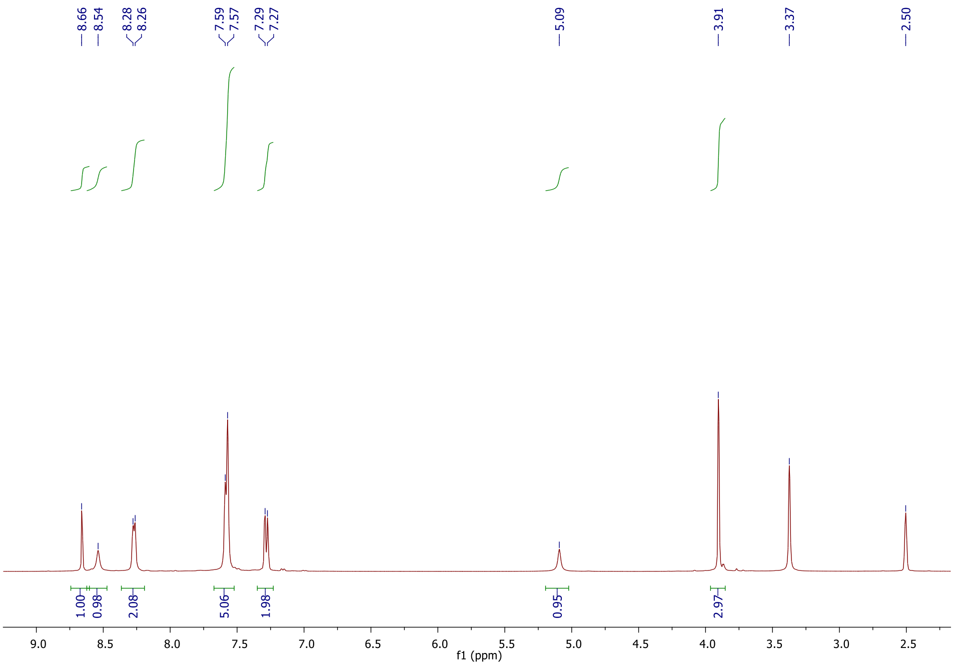

^{13}C NMR of compound **3c** (100 MHz, DMSO*d6*)

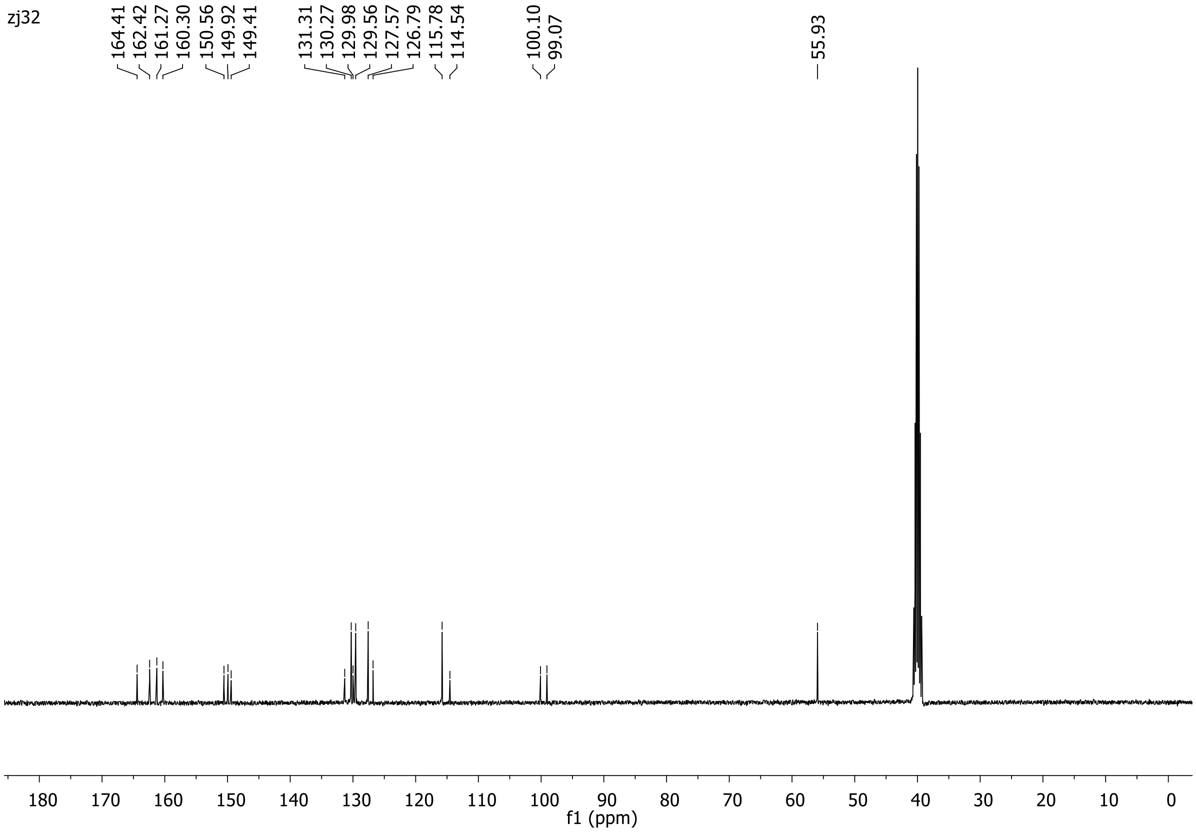

^{1}H NMR of compound **3d** (400 MHz, DMSO*d6*)

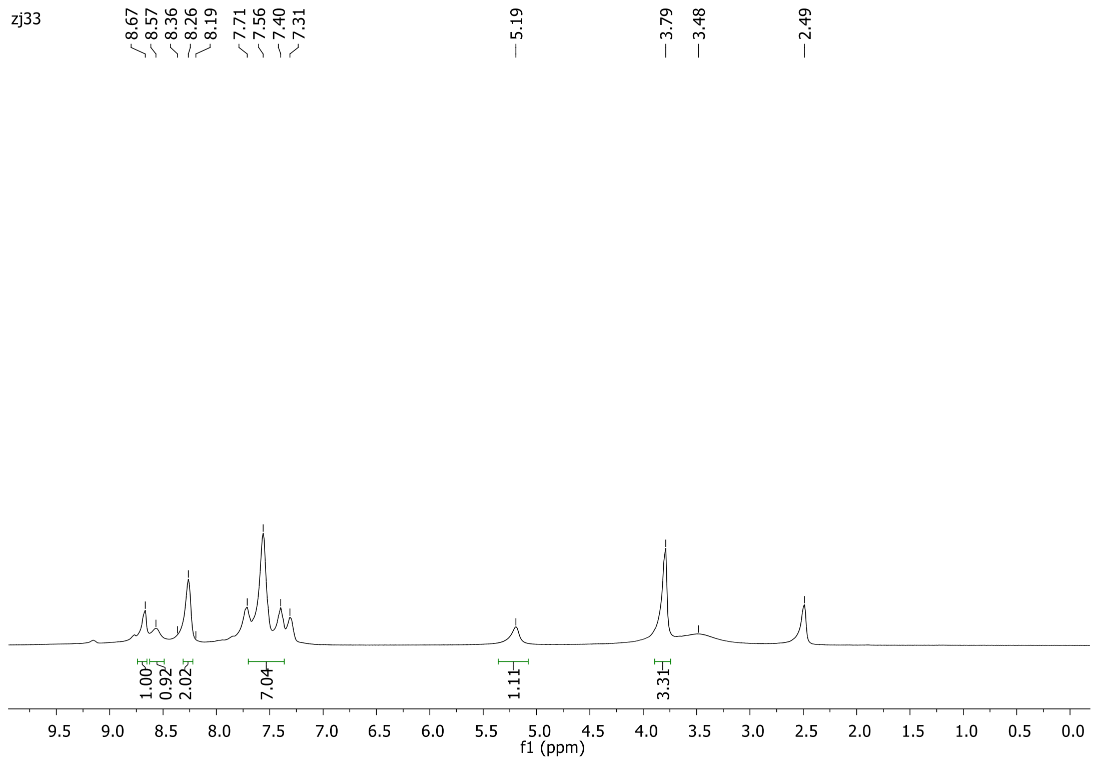

^{13}C NMR of compound **3d** (100 MHz, DMSO*d6*)

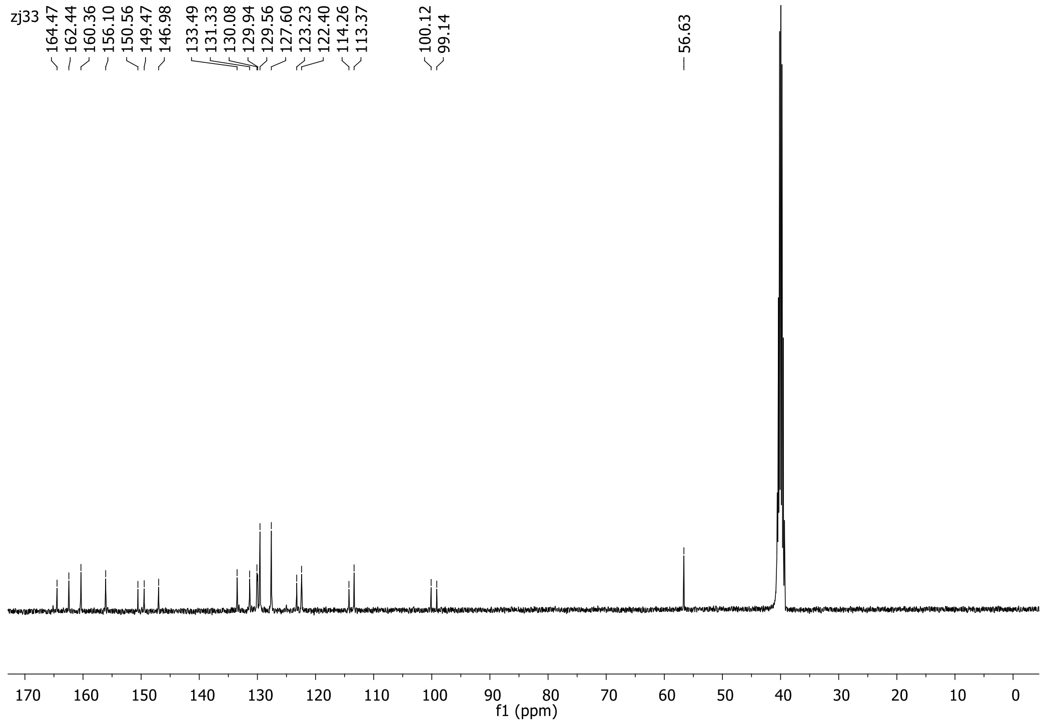

^{1}H NMR of compound **3e** (400 MHz, DMSO*d6*)

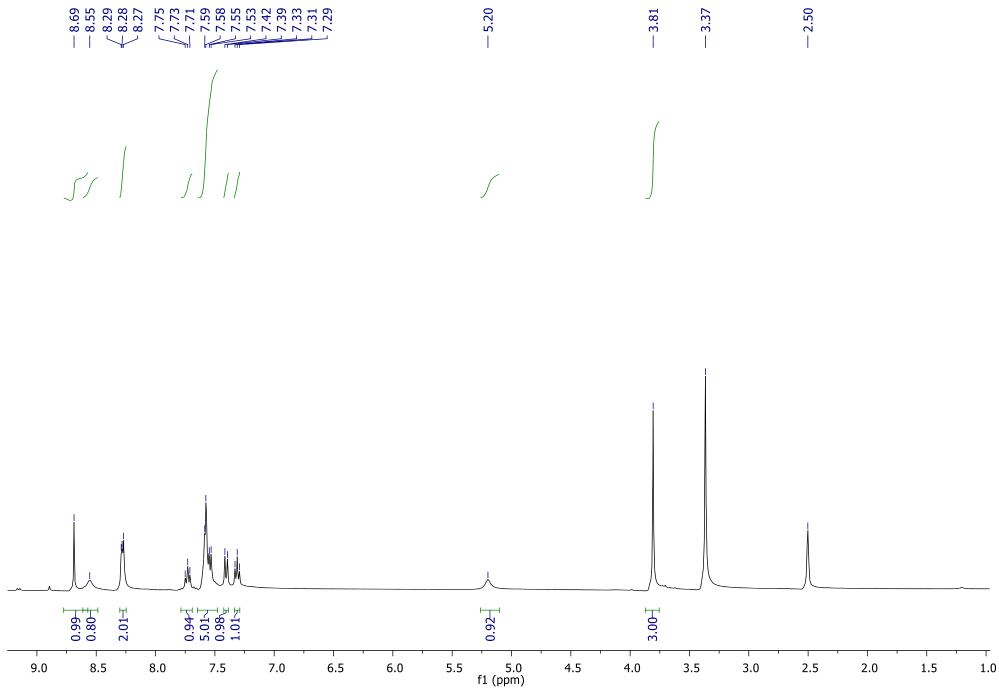

^{13}C NMR of compound **3e** (100 MHz, DMSO*d6*)

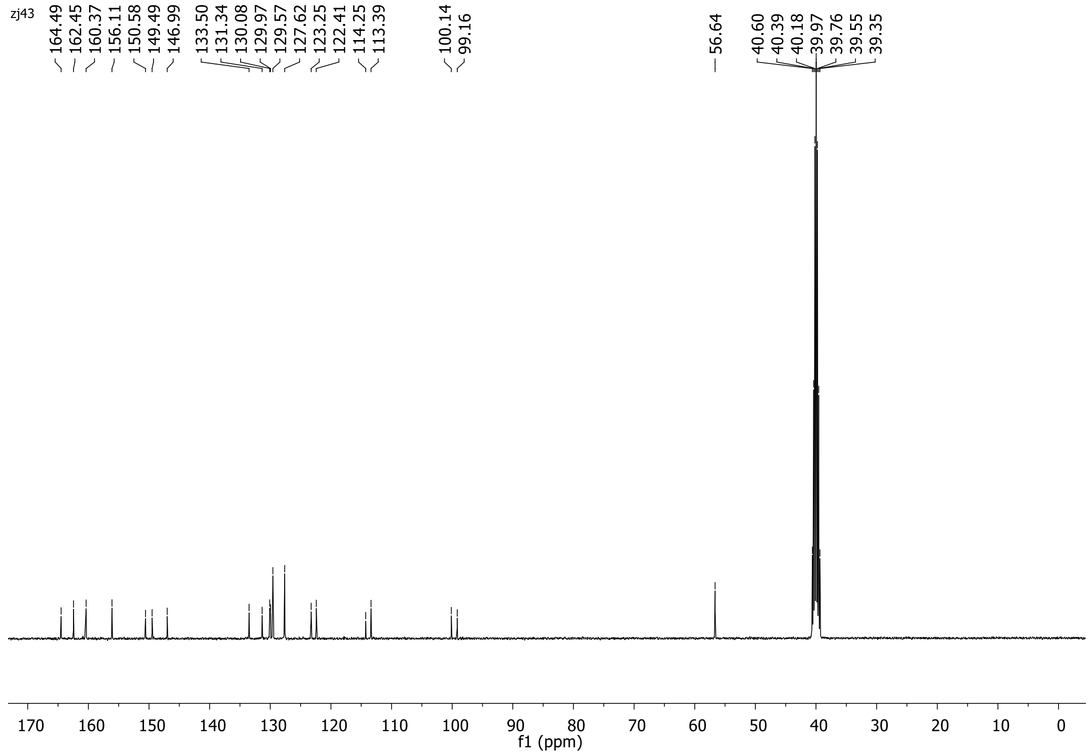

^{1}H NMR of compound **3f** (400 MHz, DMSO*d6*)

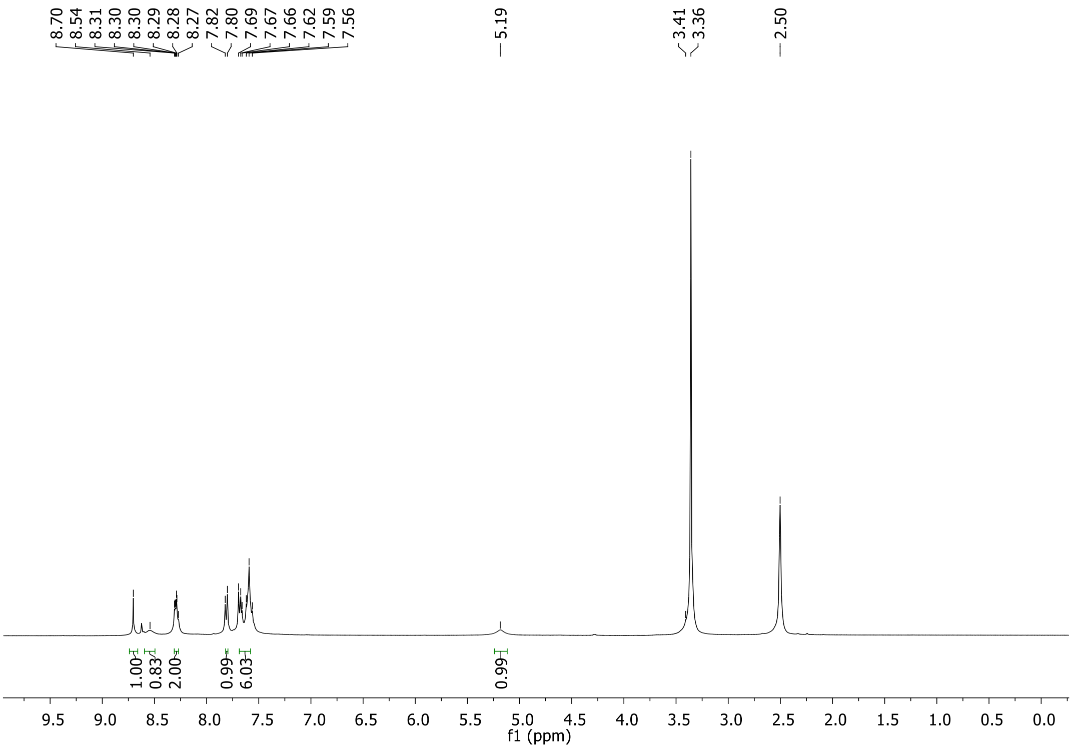

^{13}C NMR of compound **3f** (100 MHz, DMSO*d6*)

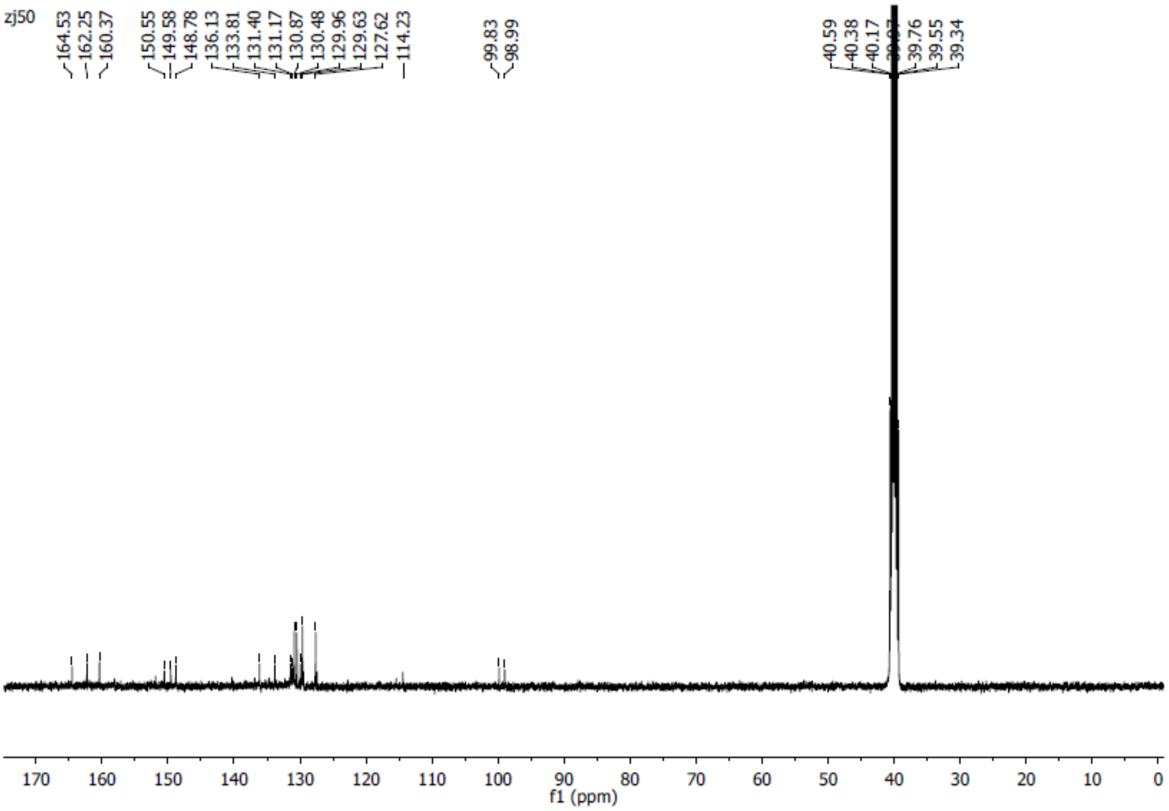

^{1}H NMR of compound **3g** (400 MHz, DMSO*d6*)

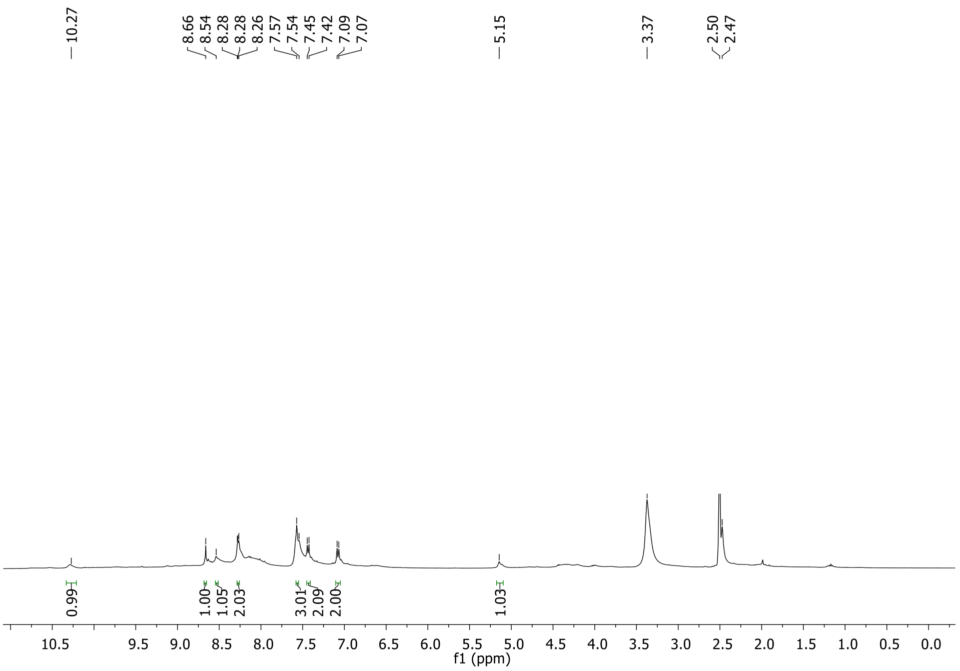

^{13}C NMR of compound **3g** (100 MHz, DMSO*d6*)

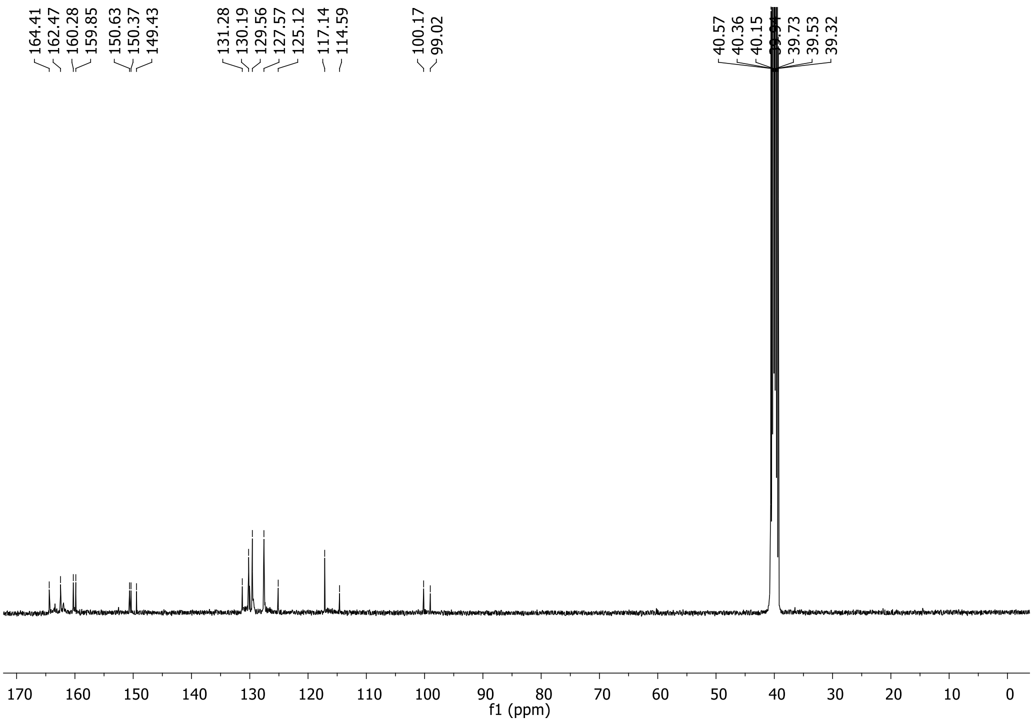

^{1}H NMR of compound **3h** (400 MHz, DMSO*d6*)

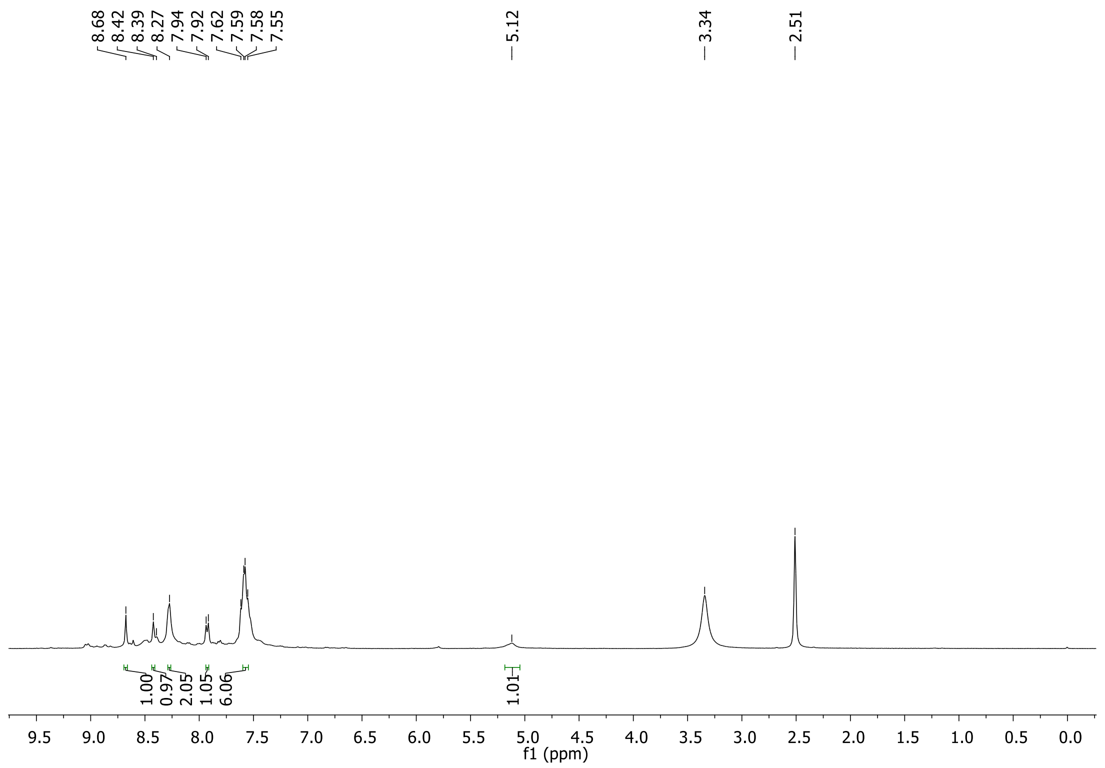

^{13}C NMR of compound **3h** (100 MHz, DMSO*d6*)

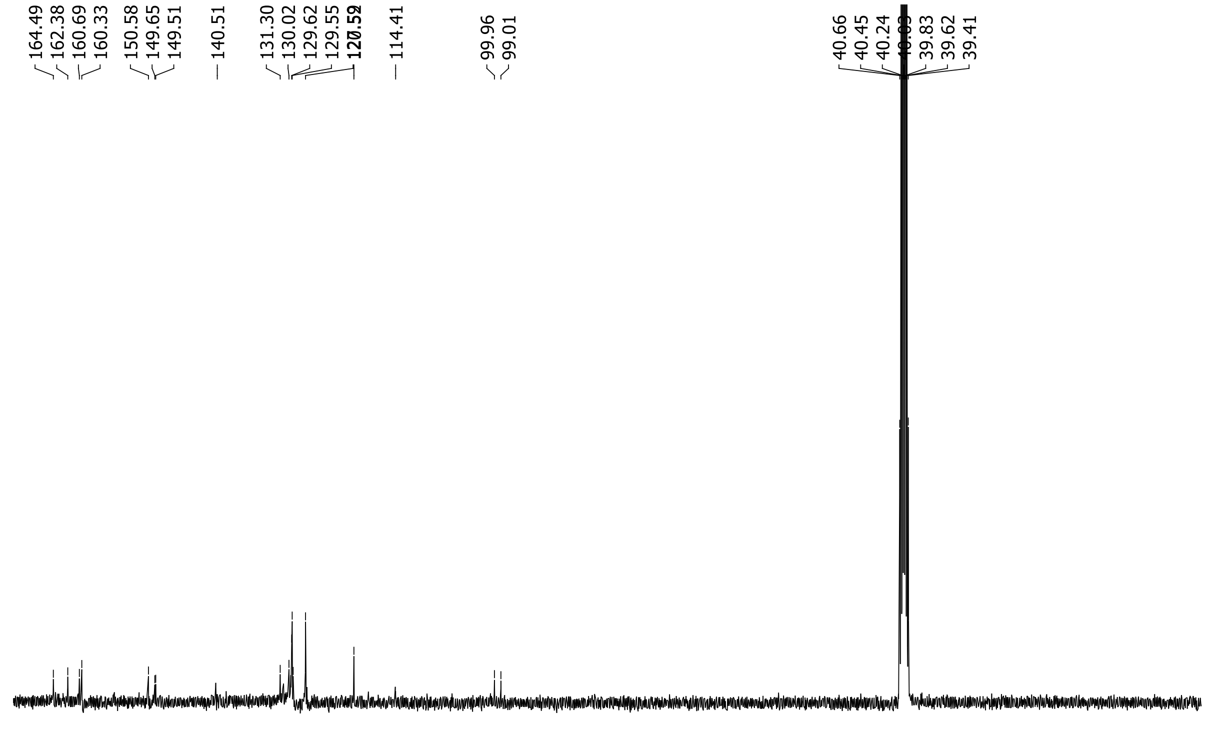

^{1}H NMR of compound **3i** (400 MHz, DMSO*d6*)

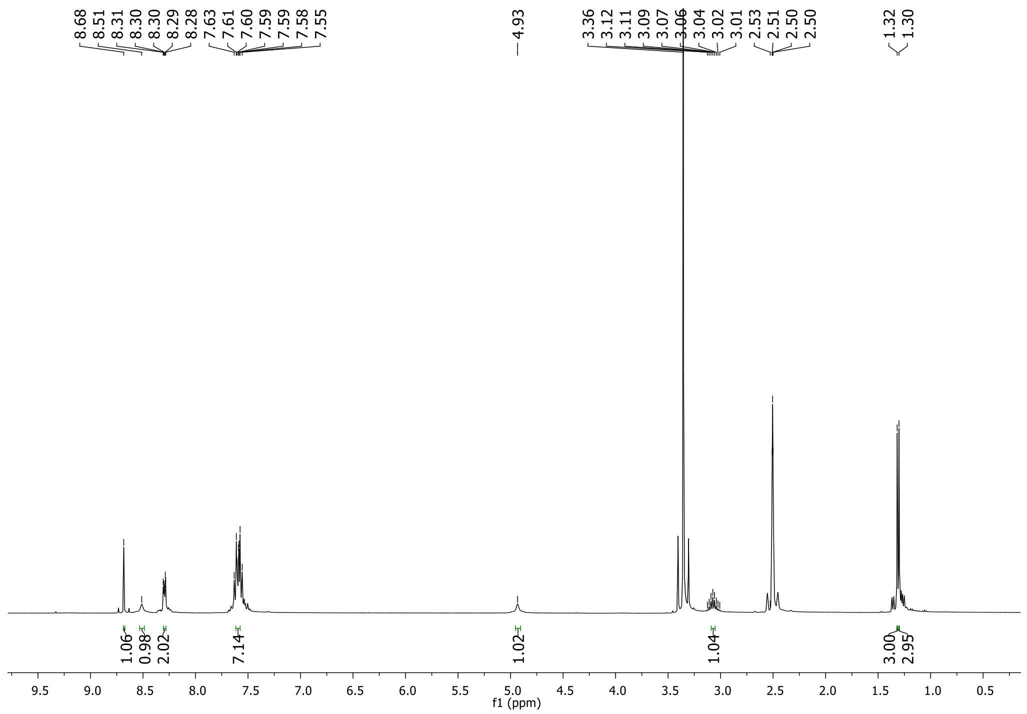

^{13}C NMR of compound **3i** (100 MHz, DMSO*d6*)

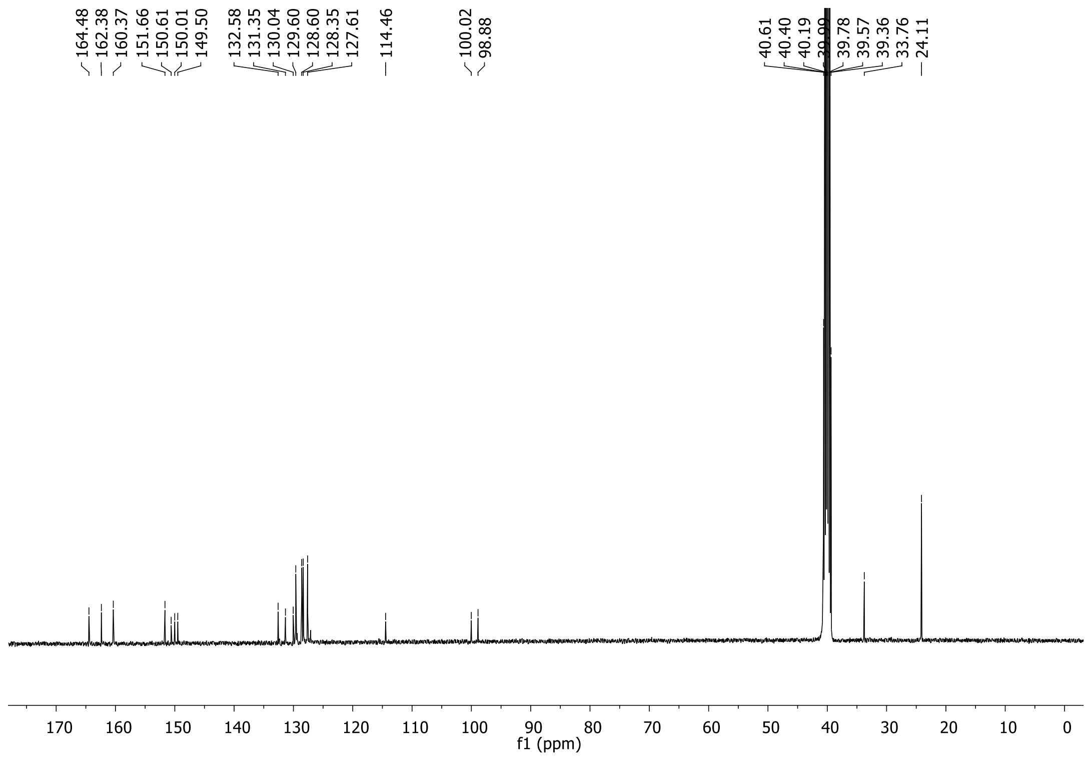

^{1}H NMR of compound **3j** (400 MHz, DMSO*d6*)

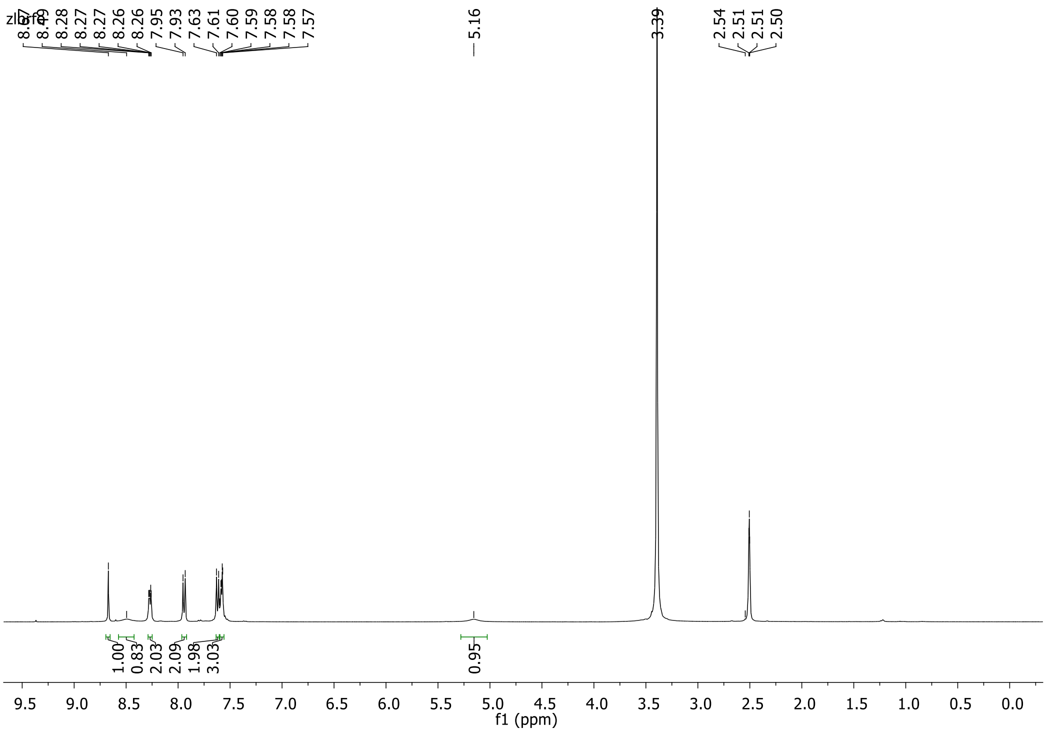

^{13}C NMR of compound **3j** (100 MHz, DMSO*d6*)

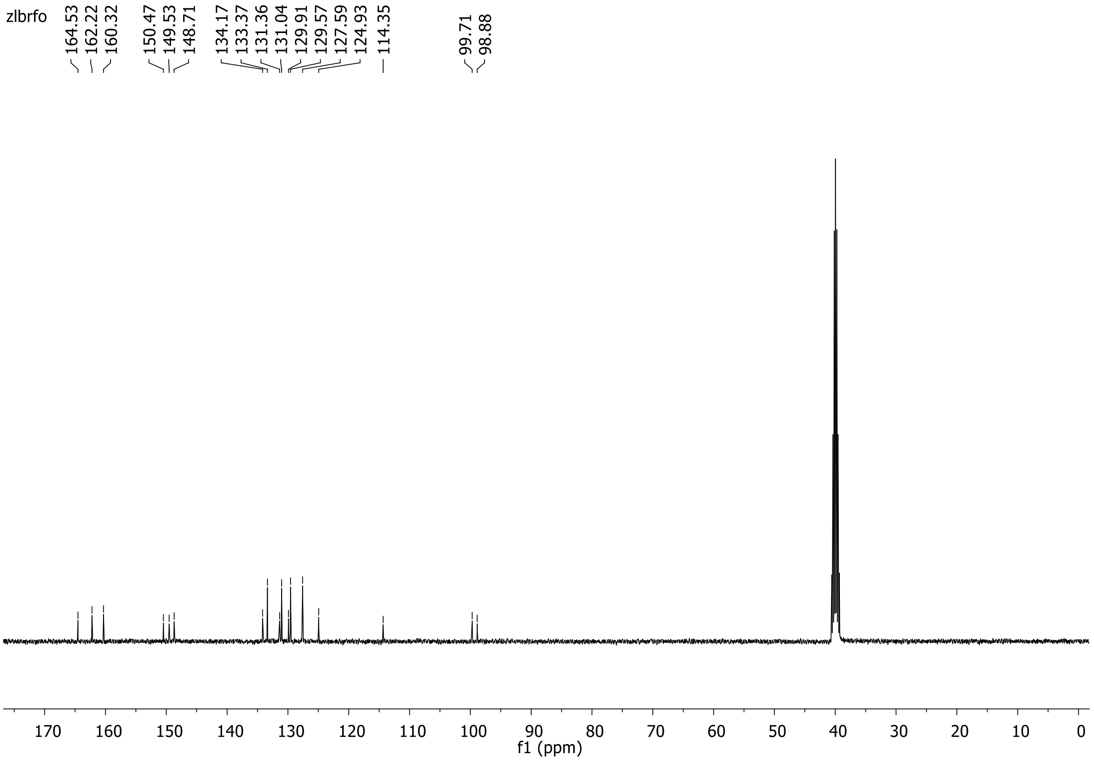

^{1}H NMR of compound **3k** (400 MHz, DMSO*d6*)

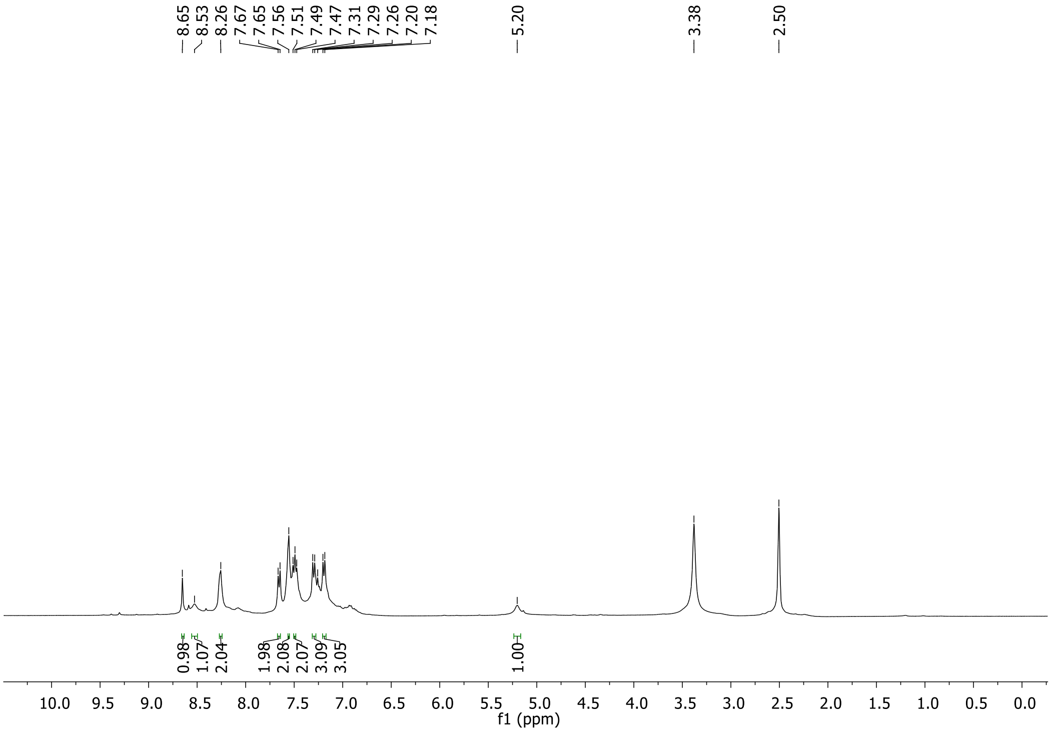

^{13}C NMR of compound **3k** (100 MHz, DMSO*d6*)

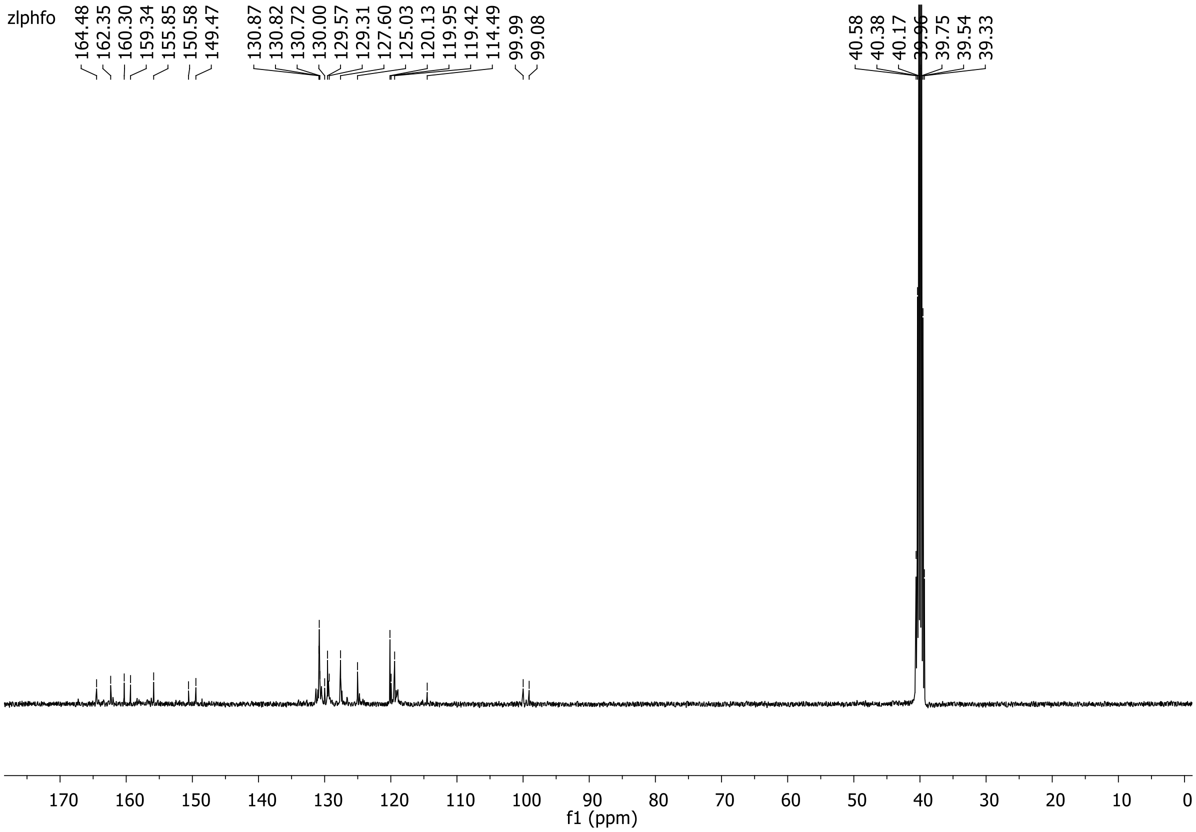

.
